# Divergent molecular pathways drive monomorphic epitheliotropic and enteropathy-associated intestinal T-cell lymphoma

**DOI:** 10.1038/s41375-025-02777-2

**Published:** 2025-10-07

**Authors:** David Vallois, Edoardo Missiaglia, Luis Veloza, Anja Fischer, Doriane Cavalieri, Vimel Rattina, Bettina Bisig, Vincent Roh, Laura Wiehle, Rita Sarkis, Emmanuel Bachy, Christophe Bonnet, Julie Bruneau, Anne Cairoli, Roland De Wind, Fanny Drieux, Romain Dubois, Jean-François Emile, Virginie Fataccioli, Kamel Laribi, Albane Ledoux-Pilon, François Lemonnier, Francisco Llamas-Gutierrez, Pierre Morel, Marie Parrens, Elsa Poullot, Leticia Quintanilla-Martinez, Jeremy Sandrini, Joan Somja, Luc Xerri, Olivier Tournilhac, Philippe Gaulard, Reiner Siebert, Laurence de Leval

**Affiliations:** 1 Institute of Pathology, Lausanne University Hospital and University of Lausanne, Lausanne, Switzerland; 2 Institute of Human Genetics, Ulm University and Ulm University Medical Center, Ulm, Germany; 3 Department of Hematology, University Hospital of Lille, Lille, France; 4 Translational Data Science Facility, AGORA Cancer Research Center, Swiss Institute of Bioinformatics, Lausanne, Switzerland; 5 Department of Hematology, Centre Hospitalier Lyon Sud and Inserm, U1111 Pierre Bénite, France; 6 Department of Hematology, University Hospital Center of Sart Tilmanand and Liège University, Liège, Belgium; 7 Department of Pathology, Necker Hospital for Sick Children, AP-HP, Paris-Cité University, Paris, France; 8 Service of Hematology, Department of Oncology, Lausanne University Hospital and Lausanne University, Lausanne, Switzerland; 9 Department of Pathology, Institute Jules Bordet, Bruxelles, Belgium; 10 Centre Henri Becquerel, Service of Anatomical and Cytological Pathology, Centre Henri Becquerel, Rouen, France; 11 Department of Pathology, University Hospital of Lille, Lille, France; 12 Department of Pathology, Ambroise Paré Hospital - Université Saint Quentin en Yvelines, Paris, France; 13 Department of Pathology, AP-HP, Henri Mondor Hospital, F-94010 Créteil, France; 14 University Paris Est Créteil, INSERM, IMRB, Créteil, France; 15 Department of Hematology, Hospital Centre Le Mans, Le Mans, France; 16 Department of Pathology, University Hospital of Clermont-Ferrand, Clermont-Ferrand, France; 17 AP-HP, Henri Mondor Hospital, Lymphoid malignancies unit, Créteil, France; 18 Department of Pathology, University Centre Hospital, Rennes, France; 19 Department of Hematology, Hospital of Lens, Lens, France and Department of Hematology, University Hospital of Amiens, Amiens, France; 20 Department of Pathology, Bordeaux University Hospital, Bordeaux University, Bordeaux, France; 21 Institute of Pathology, University Hospital Tübingen, Eberhard Karls University of Tübingen, Tübingen, Germany; 22 Department of Pathology, Le Mans Hospital Center, Le Mans, France; 23 Institut Paoli-Calmettes, CRCM and Aix-Marseille University, Marseille, France; 24 Department of Hematology, University Hospital of Clermont-Ferrand, Clermont-Ferrand, France; 25 German Center for Child and Adolescent Health (DZKJ), partner site Ulm, Ulm, Germany

**Keywords:** Oncogenesis, Cancer genomics, Cancer epigenetics, T-cell lymphoma

## Abstract

Enteropathy-associated intestinal T-cell lymphoma (EATL) and monomorphic epitheliotropic intestinal T-cell lymphoma (MEITL) have distinctive clinical context, morphology, and immunophenotype. To characterize their genetic and molecular specificities, we compared 30 EATLs and 52 MEITLs by whole-exome, RNA and miRNA sequencing and DNA methylation profiling. Highly recurrent *SETD2* loss-of-function alterations and frequent mutations of *H3-3A/B* consistently altering H3R2, implying deregulation of histone marks, were selectively found in MEITL. EATL instead harbored frequent mutations in *TET2, ARID1A*, and *KMT2D*. Highly prevalent JAK-STAT pathway mutations preferentially affected *JAK3* and *STAT5B* in MEITL, and *JAK1* and *STAT3* in EATL. Half of EATLs contained disruptive mutations in HLA class I genes, impacting class I molecule expression. EATL containing more abundant macrophages was enriched in inflammatory response signatures, with upregulation of *CD274, CXCL13*, and *IDO1* transcripts, suggesting an immunosuppressive tumor microenvironment. CpGs hypomethylated in MEITL compared to EATL were enriched in promoter regions. Unsupervised analyses of mutations, transcription, and methylation profiles concordantly segregated EATLs from MEITLs. In summary, the distinctive genetic, epigenetic, and expression footprints of EATL and MEITL established by this study expand disease-defining features, have diagnostic implications, and provide a rationale for targeted therapies.

## Introduction

Enteropathy-associated T-cell lymphoma (EATL) and monomorphic epitheliotropic intestinal T-cell lymphoma (MEITL) are rare malignancies derived from intestinal intraepithelial T lymphocytes, formerly considered as two disease subtypes, namely EATL type I and type II [1–4]. EATL, a rare complication of celiac disease (CeD), is more prevalent in northern Europe and North America, and MEITL, reportedly lacking association with CeD, is the most prevalent intestinal T-cell lymphoma in Asia [5]. Both usually occur in older adults, present as tumors or ulcers in the small intestine, are revealed by (sub)acute abdominal symptoms often requiring surgery, and are associated with an unfavorable prognosis [5–7]. Morphologically, EATL tends to be pleomorphic, while MEITL usually consists of monomorphic cells and lacks significant inflammation, although a pathological spectrum including atypical morphology is now recognized [7, 8]. Besides a common cytotoxic T-cell phenotype, coexpression of CD8 and CD56 is characteristic of MEITL [4], which typically lacks CD30 expression, often reported in EATL [2, 3].

At the genetic level, earlier studies found shared and distinct chromosomal imbalances [9–12]. More recently, high-throughput sequencing studies have generated mutation maps, with MEITL more extensively analyzed. Our group discovered highly recurrent disruptive alterations of *SETD2* in MEITL in patients from Western Europe [13], a finding confirmed in studies from Asia and America [8, 14, 15]. Other recurrently altered genes in MEITL include *STAT5B, JAK3* and *TP53* [7, 13, 16–18]. In EATL, where available data is limited, the potential implication of *SETD2* is controversial, and overlap in mutational portrait has been reported with both MEITL and type II refractory CeD, notably in *JAK1* (at the p.G1097 hotspot) and *STAT3* [13, 14, 19].

Our previous research on MEITL has uncovered its genetic portrait and pathological spectrum and identified molecular biomarkers influencing patients’ outcomes [7]. In this study, we aimed to establish and clarify the similar and diverging features of EATL. Having assembled a large cohort of 82 patients with EATL or MEITL, we performed a multimodal analysis integrating whole-exome and RNA sequencing, and DNA methylation and miRNA profiling.

## Material and methods

The flow chart of the study is described in Fig. S1

### Cases studied

Eighty-two patients diagnosed with EATL or MEITL and available formalin-fixed paraffin-embedded (FFPE) tissue were subjected to an integrative pluri-omics analysis. Cases were centrally reviewed (LV, LdL, PG) according to the revised 4th WHO classification criteria [1]. The study protocol was approved by the Commission cantonale d’éthique de la recherche sur l’être humain (CER-VD, protocol 382/14), the Comité de Protection des Personnes-Ile-de-France IX (CPP08/009), and the Ethical Committee of the University of Tübingen (105/2013BO2), and the study was conducted in accordance with the Declaration of Helsinki. Informed consent from the patients was obtained in accordance with the respective regulations of the Ethical Committees. Clinicopathological features of the patients are summarized in Table 1. Refer to Supplementary methods for details on histology, immunohistochemistry, and fluorescence in situ hybridization.

**Table 1:** Summary of clinical and pathological features of 82 patients with monomorphic epitheliotropic intestinal T-cell lymphoma (MEITL) or enteropathy-associated T-cell lymphoma (EATL).

	MEITL (*n* = 52)	EATL (*n* = 30)	*p* value
**Clinical features**			
Reported celiac disease	0/52 (0%)	13/30 (43%)	**<0.005**
Age, median, years (range)	67 (29-91)	64 (34-86)	0.37
Gender (male/female)	27:25	19:11	0.2
Acute event at presentation	38/44 (86%)	12/18 (67%)	0.09
Bowel perforation	33/44 (75%)	8/18 (44%)	**0.037**
Bowel obstruction	12/44 (27%)	6/18 (33%)	0.76
Lugano stage			
Stage I/II	27/44 (61%)	8/13 (61%)	1.000
Stage III/IV	17/44 (39%)	5/13 (39%)	
PS			
0-1	18/40 (45%)	4/8 (50%)	1.000
≥2	22/40 (55%)	4/8 (50%)	
**Morphology**			
Typical	31/52 (60%)		
Atypical	21/52 (40%)		
Pleomorphic	13/52 (25%)	30/30 (100%)	**<0.005**
Anaplastic	1/52 (2%)	15/30 (50%)	**<0.005**
Necrosis	7/52 (13%)	22/30 (73%)	**<0.005**
Starry-sky	5/52 (10%)	3/30 (10%)	1.000
Angiotropism	14/51 (27%)	19/29 (65%)	**<0.005**
Epitheliotropism	36/41 (88%)	2/20 (10%)	**<0.005**
Moderate/abundant inflammation	3/52 (6%)	20/30 (67%)	
**Immunophenotype**			
CD8	47/52 (90%)	4/30 (13%)	**<0.005**
CD56	46/52 (88%)	2/28 (7%)	**<0.005**
CD3	52/52 (100%)	27/30 (90%)	**0.046**
CD2	20/47 (42%)	12/24 (50%)	0.619
CD5	2/51 (4%)	2/30 (7%)	0.624
CD7	48/48 (100%)	22/23 (96%)	0.324
CD4	2/52 (4%)	2/27 (7%)	0.603
CD103	40/51 (78%)	15/25 (60%)	0.108
CD30	0/47 (0%)	25/29 (86%)	**<0.005**
TIA1	47/52 (90%)	20/25 (80%)	0.108
Granzyme B	37/52 (71%)	19/28 (68%)	0.596
Perforin	27/52 (52%)	22/29 (76%)	**<0.005**
CD20	9/49 (18%)	1/29 (3%)	**0.046**
CD79a	3/41 (7%)	0/10 (0%)	0.567
**TCR**			
TCRβ	17/50 (34%)	8/28 (29%)	0.472
TCRγδ	26/50 (52%)	8/27 (30%)	**0.050**
TCRαβ-TCRγδ +	22/49 (44%)	4/26 (15%)	**0.012**
TCRαβ + TCRγδ-	12/49 (24%)	4/26 (15%)	0.391
TCRαβ + TCRγδ +	4/49 (8%)	3/26 (11%)	0.685
TCRαβ-TCRγδ-	11/49 (22%)	15/26 (58%)	**0.004**

Statistically significantly *p*-values (≤0.05) are in bold font.

### Whole-exome sequencing (WES)

WES of 14 MEITLs was previously reported [13]. Twenty-six EATLs and 23 additional MEITLs (including 5 with only tumoral tissue available) were sequenced using the xGen research panels v1.0 and v2.0 (IDT DNA, Newark, New Jersey, USA). Refer to Supplementary methods for details on DNA extraction, WES procedure, and analysis.

### RNA sequencing (RNA seq)

Libraries from 48 MEITLs and 27 EATLs were prepared starting from 1 µg of total RNA using the TruSeq Stranded total RNA Gold kit (Illumina, San Diego, California, USA) and were sequenced on a HiSeq 4000 machine (Illumina). Refer to Supplementary methods for details on RNA extraction, RNAseq library preparation and sequencing, differential expression and deconvolution analyses, determination of HLA haplotypes, and miRNA profiling.

### Genome-wide DNA methylation profiling

Genomic DNA was bisulfite-converted using the EZ DNA Methylation kit (ZymoResearch) according to manufacturer’s instructions. DNA methylation profiles were generated using the Infinium MethylationEPIC BeadChip (Illumina). Refer to Supplementary methods for details on methylome, differentially methylated loci, chromatin state enrichment and deconvolution analyses.

### Integrative analyses

Methodology of integrative analyses of RNAseq data, with WES findings, methylome, and microRNA profiles is described in the Supplementary methods.

### Statistical analyses

The Kaplan-Meier method was used to summarize survival outcomes and compared by the log-rank test and two-sided 95% confidence intervals (survival (v.3.8.3) and survminer (v.0.5) packages in R. Overall survival data were collected considering time from first diagnosis to time of last event (death or last follow-up).

Categorical variables were compared using Fisher’s exact or chi-square test, while continuous variables were analyzed using the nonparametric Mann–Whitney U test, if not otherwise specified. Multiple testing adjustments were performed by applying the Benjamini-Hochberg procedure.

## Results

### Patients’ characteristics, tumor histopathology, and disease course

The clinical characteristics of the study population are summarized in Table 1. Thirteen patients with EATL (43%) had a previous diagnosis of CeD. HLA class II genes typing was derived from RNA sequencing data, focusing on the HLA-DQA1 and HLA-DQB1 genes encoding the HLA-DQ heterodimer, the main genetic predisposing factor to CeD [20, 21]. Most patients with EATL (23/27) carried the high-risk heterodimer HLA-DQ2.5, none carried HLA-DQ8, and three carried haplotypes associated with a lower risk of CeD. The frequencies of the high-risk haplotypes in MEITL patients did not differ from the prevalence observed in general Caucasian populations (Fig. S2). More patients with MEITL (33/44, 75%) than EATL (8/18, 44%) presented with bowel perforation (*p* = 0.037), and most patients (61%) had stage I-II disease.

**Fig. 1:**
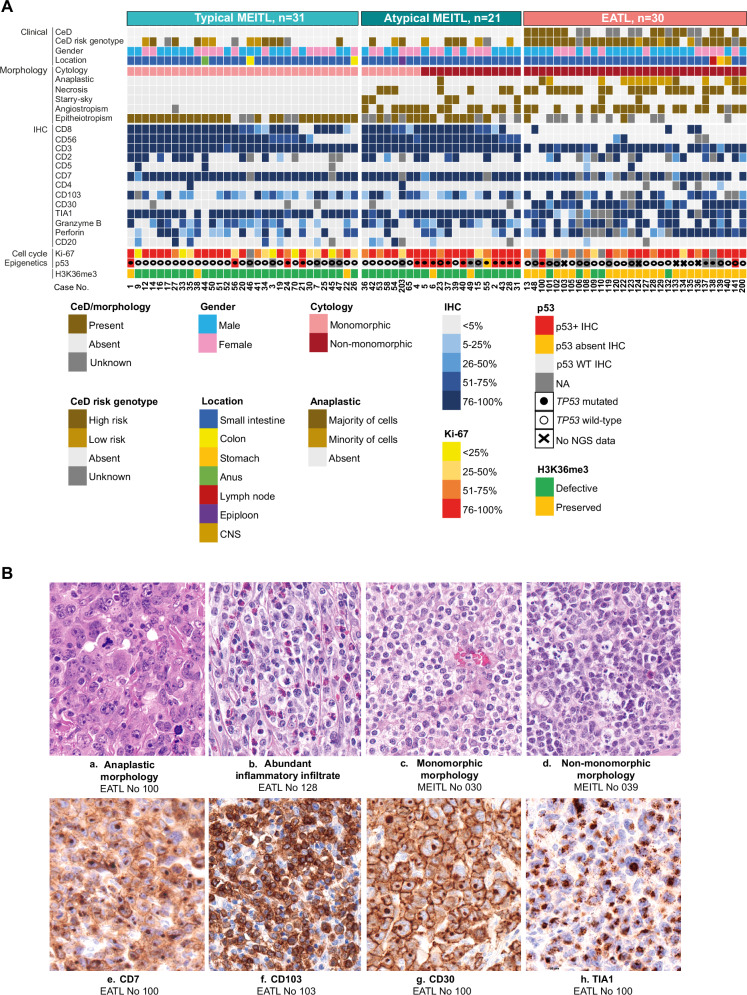
Clinico-pathological features of the study cohort. **A** Clinical characteristics and histopathology features of tumors in 82 patients with monomorphic epitheliotropic intestinal T-cell lymphoma (MEITL) or enteropathy-associated T-cell lymphoma (EATL); CNS central nervous system, IHC immunohistochemistry, NGS next-generation sequencing. **B** Representative illustrations of morphology and immunophenotype (H&E and immunoperoxidase, 400X).

Pathological features are shown in Table 1 and Fig. 1. All EATLs were composed of pleomorphic large cells, with frequent anaplastic morphology (50%), angioinvasion and/or angiodestruction (65%), necrosis (73%), and moderate to abundant inflammation (67%) (Fig. 1B-a, b). MEITLs comprised 31 tumors with typical histology (60%) and 21 atypical cases [7] with non-monomorphic, starry-sky pattern, necrosis, or angioinvasion (Fig. 1B, c, d). Expression of pan-T-cell antigens, lack of CD5, and expression of CD103 were overall similar in both entities (Fig. 1B–e, f). Expression of CD3 was occasionally lost in EATL (3/30 cases). Positivity for CD8 (90%) and CD56 (88%) in MEITL contrasted with rare expression in EATLs (13% and 7%, respectively) (*p* < 0.005). Most EATLs were CD30-positive (86%) (Fig. 1B–g), while all MEITLs were CD30-negative (*p* < 0.005). Except for one MEITL, all cases (81/82) had a cytotoxic immunophenotype (Fig. 1B–h), with expression of perforin more frequent in EATL than MEITL (76% versus 52%, *p* < 0.005). Aberrant coexpression of CD20 was observed in 9/49 MEITLs versus 1/29 EATLs (*p* = 0.046). Silencing of TCR expression was more common in EATL than MEITL (58% versus 22%, *p* = 0.004), and MEITLs were more often positive for TCRγδ (52%) than TCRαβ (34%).

After a median follow-up of 46 months (alive patients), the median overall survival (OS) of 24 patients with EATL was 7.9 months (range 0.1–187.4), and that of 45 patients with MEITL was 5.8 months (range 0.03–71) (*p* = 0.17) (Fig. S3A). For patients with MEITL, performance status was the only clinical factor linked to OS (Fig. S3B). No associations were found with CeD or anaplastic histology in EATL (Fig. S3C).

### Mostly non-overlapping driving mutations in MEITL and EATL

By WES, a total of 7101 non-synonymous somatic mutations (Table S1) were found in 26 EATLs and 37 MEITLs (5344 missense, 904 splice site, 473 frameshift, 290 nonsense, 72 inframe_indel, 9 start lost and 9 stop lost), corresponding to a median mutation count of 91 (range 4–254) and 72 (41–839), respectively. The overall median tumor mutational burden was 2 non-synonymous mutations per Mb; with no significant difference between EATL (2.4; range: 0.1–6.9) and MEITL (1.9; range: 1–17.1). No differences were observed either in mutation types or mutational signatures (not shown).

**Fig. 2:**
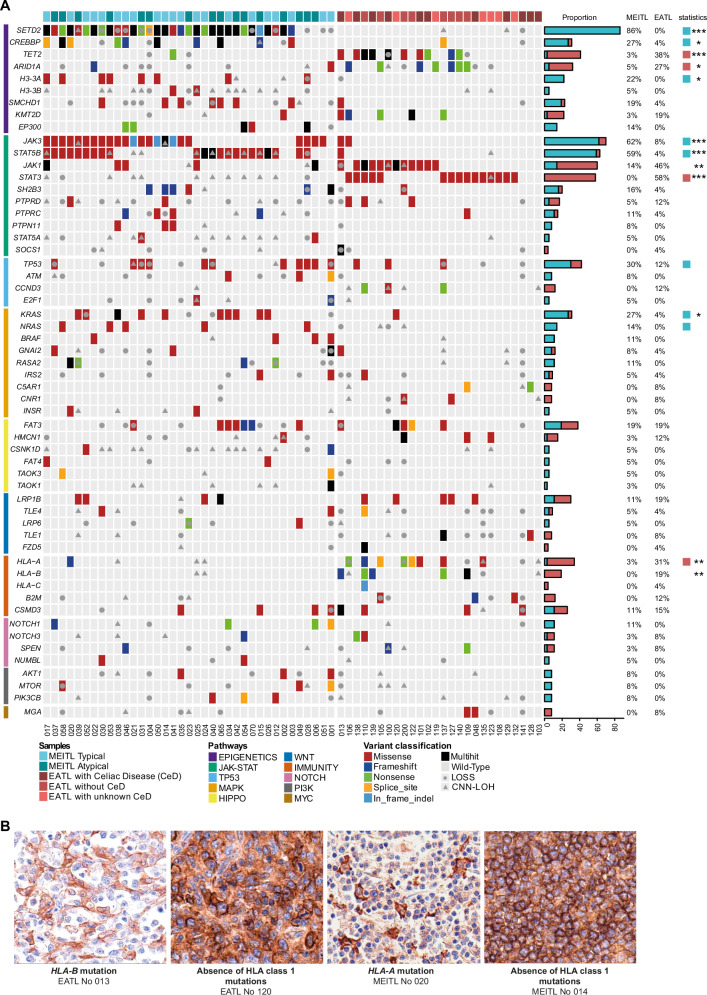
Genetic alterations. **A** Genomic alterations landscape of selected genes grouped by pathways in 37 MEITLs and 26 EATLs. Individual patients are displayed as columns, and mutation types are color-coded. Mutation frequencies in MEITL and EATL, significant differences (*p*-values, Fisher test, *p < 0.05, **p < 0.01, ***p < 0.001), and results of MutSig2CV analysis (colored squares) are shown on the right side. Genes mutated at least three times in the cohort were selected for representation; in addition, a few additional genes less frequently mutated but functionally related to the latter, were also listed. The complete list of mutations is available in Supplementary Table S1. **B** Representative immunostainings of HLA-I protein in EATL and MEITL with or without HLA class I gene mutation.

Overall, the highest frequencies of mutations were observed among genes involved in epigenetic regulation (49/63, 78%) and JAK-STAT pathways (58/63, 92%). However, the impacted genes and their mutation prevalence varied greatly between the two entities (Fig. 2 and S4A).

Mutations, mainly loss-of-function, of the histone-lysine N-methyltransferase *SETD2* gene were present exclusively in MEITL (32/37 cases; 86%). In EATL, alterations in *SETD2* were limited to a few broader genomic events, including copy number neutral loss of heterozygosity (CNN-LOH) in two cases and a heterozygous deletion in one case. Conversely, loss-of-function mutations, such as frameshifts or stop-gains, in *TET2* and *ARID1A* were observed in 10/26 (38%) and 7/26 (27%) EATLs and in only 1/37 (3%) and 2/37 (5%) MEITLs, respectively. Among other epigenetic modifiers, deleterious mutations of the histone acetyltransferase *CREBBP* were prevalent in MEITL (10/37; 27%) and rare in EATL (1/26 4%). Moreover, our study highlighted three epigenetic regulation-related genes altered only in MEITL: *H3-3A* (8/37; 22%), *H3-3B* (2/37; 5%), and *SMCHD1* (7/37; 19%) (Fig. S4B). Notably, all mutations in *H3-3A* and *H3-3B*, which encode identical H3.3 histone protein [22], affected the same arginine H3R2 at position 2 of the protein (R2C/H/Q) and were mutually exclusive. The missense mutations found in *SMCHD1* (structural maintenance of chromosomes flexible hinge domain containing 1), a gene involved in the regulation of chromatin structure and DNA methylation [23], were distributed along its coding sequence.

Mutations in genes related to the JAK/STAT pathway, very frequently involved *JAK3* (23/37; 62%) and *STAT5B* (22/37; 59%) in MEITL. Only two EATL cases harbored mutations in *JAK3* or* STAT5B*. In contrast, EATL had recurrent mutations in *STAT3* (15/26; 56%) and *JAK1* (12/26; 46%), which were absent (*STAT3*) or rare (*JAK1*, 5/37; 14%) in MEITL. Thus, considering the JAK-STAT pathway, there was a highly significant difference in mutation patterns in EATLs and MEITLs (p < 0.0001). All mutations in *STAT3* and *JAK1* were missense, most of them characterized as activating [19, 24–26]. The *JAK1* mutations in EATL clustered in the G1097 hotspot of the protein kinase 2 domain, but those in MEITL had a different distribution (Fig. S4B).

Mutations in *TP53* almost exclusively affecting the DNA-binding domain (Fig. S4B) were more prevalent in MEITL (11/37, more often in atypical cases, *p* < 0.05) than in EATL (3/26) (p = 0.13) (Fig. 2A).

Mutations impacting the MAPK pathway were recurrent in MEITL and sporadic in EATL. *KRAS* and *NRAS* were mainly altered by mutually exclusive canonical hotspots of missense mutations described in carcinomas [27]. Missense mutations in *GNAI2*, coding for a G-alpha protein involved in G-protein-coupled receptor signaling pathway and a negative regulator of the MAPK pathway, reported previously in MEITL [17], were found in 3/37 (8%) MEITLs and 1/26 (4%) EATLs.

Interestingly, 13/26 EATLs (50%) harbored non mutually exclusive disruptive mutations in HLA class I genes *HLA-A, HLA-B, HLA-C*, or *B2M*. Mutations in *HLA-A* were more frequent in patients with a history of CeD (*p* < 0.05). By immunohistochemistry, expression of HLA class I proteins (Fig. 2B) was reduced or absent in 12/13 EATLs with HLA class I gene mutations and preserved in 9/13 EATLs with wild-type HLA class I genes (*p* = 0.001). Among MEITLs, only one case harbored an *HLA-A* mutation and was negative for HLA class I immunostaining, while 25/27 cases with WT class I genes had preserved HLA class I expression.

The most significantly mutated genes by MutSig2CV algorithm (q val<0.01), were *SETD2*, *STAT5B, NRAS, TP53, KRAS, H3-3A, CD53, JAK3* and *CREBBP* in MEITL, and *HLA-A, STAT3, ARID1A*, and *TET2* in EATL (Fig. 2A and Table S,2). With respect to disease subgroups, *KRAS* and *TP53* were identified as drivers in atypical MEITL, *JAK1* in EATL with CeD, and *HLA-A* was close to significance in EATL with no CeD history (q val = 0.019). OS in both EATL and MEITL was not significantly influenced by the presence of main driver mutations (Figs. S5 and S6).

Then, we explored the proportion of transcripts carrying mutations using parallel WES and RNAseq data available in 59 cases (Fig. S7). Overall, focusing on 5396 non-synonymous single-nucleotide variants (SNVs) detected by WES, 1091 (20%) had reads with only wild-type sequence, 1510 (28%) both mutant and wild-type, and 147 (3%) exclusively mutated reads. Thus, only 31% of the DNA-based mutations were detected in the RNA transcripts, which is in line with a previous pan-cancer study [28]. The driver mutations, including those in *STAT5B, JAK3*, *SETD2* and *JAK1* genes, were proportionally more often transcribed when compared to the overall distribution (Table S,3).

### Higher chromosomal instability in MEITL

**Fig. 3:**
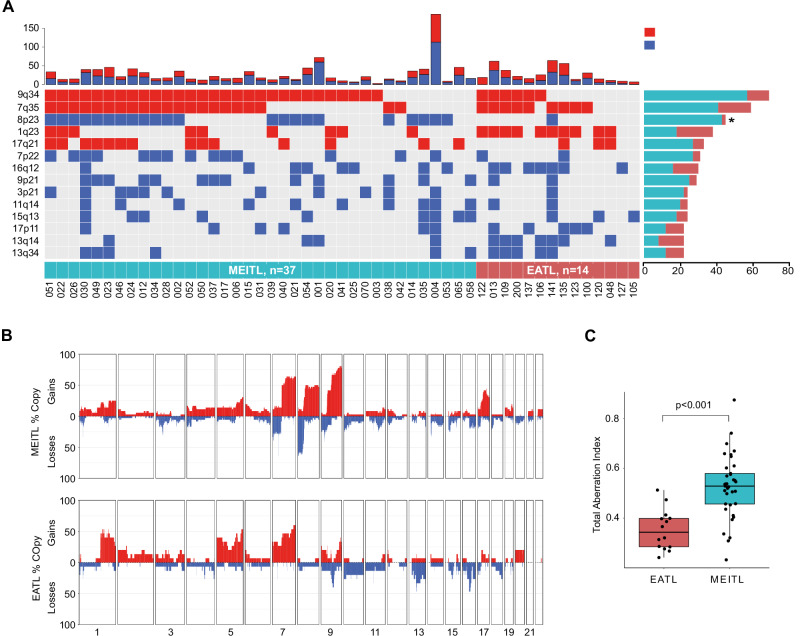
Copy number variations. **A** The alterations in individual cases (37 MEITLs and 14 EATLs displayed as columns) are shown, total number of CNVs as bars in the upper part, and the 14 significantly altered CNVs based on GISTIC analysis and ranked by decreasing overall frequency in the lower part, with their proportions shown on the right side (*p < 0.05, chi-squared test, and the p-value corrected for multiple testing using Benjamini and Hochberg False Discovery Rate.) **B** Penetrance plots showing the frequency of gains (red) and losses (blue) across the genome of MEITLs and EATLs. **C** Boxplot of genomic instability measured by total aberration index. Differences between groups were assessed by the Mann-Whitney U test (*P* values shown).

Copy Number Variations (CNVs) profiles were derived from WES from 14 EATLs and 37 MEITLs with ≥40% neoplastic cell content. GISTIC analysis of these 51 cases identified 14 chromosomal regions significantly gained or deleted, although at different frequencies in EATL and MEITL (Fig. 3A, B). Consistent with previous findings [9–12], we observed gains in 9q (78% versus 33%, *p* = 0.06) and 1q (22% versus 53%, *p* = 0.22), and losses in 16q (19% versus 40%, *p* = 0.45) in both MEITL and EATL, respectively. Losses in the 8p23.1 region, which contains multiple genes related to immune function, T-cell regulation (*TNKS, PPP2CB*,) and tumor suppression (*CSMD1, MSR*), were enriched in MEITL (61% versus 7%, *p* = 0.02). Considering EATL and MEITL separately, significant CNVs were identified only in MEITL (Fig. S8), These included 9 focal deletion peaks, notably in 3p21 (11/37; 29.7%), 9p21 (13/37; 35.1%) and 17p13 (7/37; 18.9%), and four focal gains peaks in 7q36 (22/37; 59.5%), 8q12 (17/37; 46%), 9q34 (29/37; 78.4%) and 17q22 (15/37; 40.5%). Overall, MEITL showed higher chromosomal instability as measured by the total aberration index (*p* < 0.001) (Fig. 3C).

### Distinctive transcription profiles

**Fig. 4:**
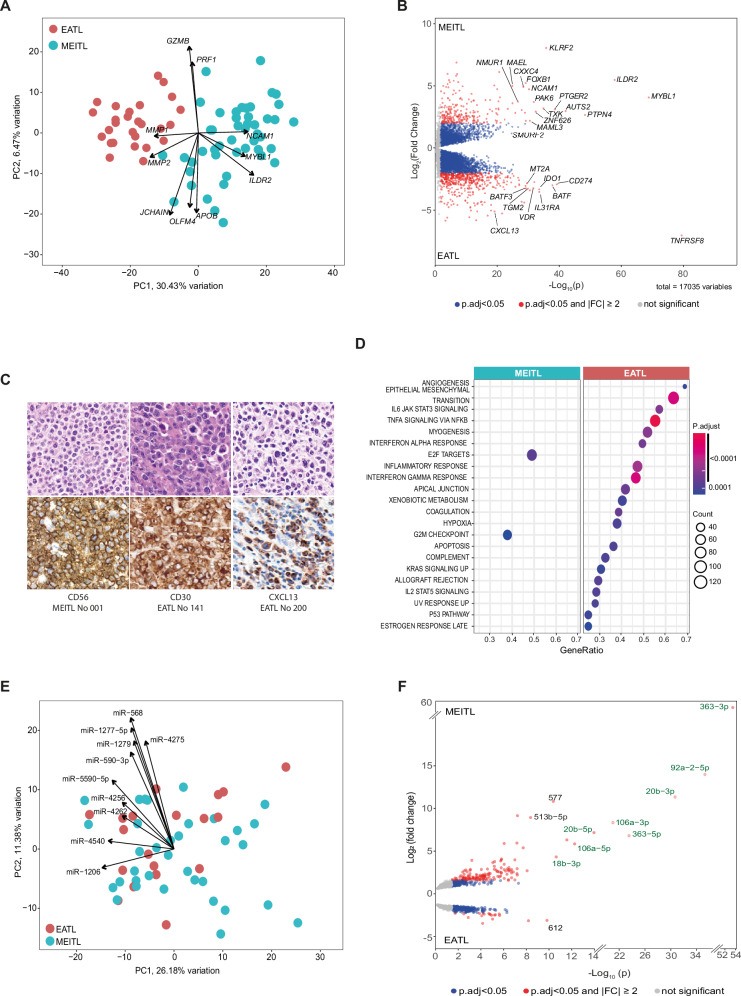
Transcriptome (genes and miRNAs) expression profiles. **A** Principal component analysis (PCA) of gene expression profiles of 48 MEITLs and 27 EATLs, considering the top 2% most variable genes. The first principal component is on the x-axis, and the second on the y-axis. PC1 accounts for 30.4% of the observed variance. Arrows indicate the expressed genes driving samples distribution, and which direction, and weight. **B** Volcano plot representation of differential gene expression. The y-axis shows the log2-transformed ratio of mean expression in MEITL over mean expression in EATL. The x-axis shows the -log10 transformed p-value (after Benjamini–Hochberg correction) of a Wald test between MEITL and EATL. Differential expression analysis found 3139 significantly upregulated genes in MEITL, and 4831 in EATL, of which 840 and 2067 with a minimal 2-fold change and adjusted *p* value < 0.05. Each point is one gene, those differentially expressed (adj. *p* value < 0.05) colored in blue and red according to fold change ≤2 or ≥2x, respectively. **C** Representative immunostainings of markers differentially expressed in EATL and MEITL. **D** Gene Enrichment Analysis using hallmark gene sets from the Molecular Signatures Database (MSigDB). The plot shows the signatures that were found significantly differentially enriched between MEITL and EATL with an adjusted *p* value ≤ 0.01 (corrected for multiple testing using Benjamini and Hochberg). Gene Ratio is the ratio of the number of enrichment genes versus the number of genes in the pathway/signature. **E** Principal Component Analysis of miRNA expression profiles does not segregate MEITL (*n* = 35) and EATL (*n* = 19) cases. Arrows indicate the expressed miRNAs driving samples distribution, and which direction, and weight. **F** Volcano plot showing 593 miRNAs differentially expressed in MEITL and EATL. The x-axis shows the -log10 transformed p-value (after Benjamini–Hochberg correction) of a Wald test between MEITL and EATL. Each point is one gene, those differentially expressed (adj. *p* value < 0.05) colored in blue and red according to fold change ≤2 or ≥2x, respectively. Green color denotes members of the miR-106a-363 cluster.

Using transcriptome sequencing data and unsupervised dimensionality reduction algorithms or hierarchical clustering, we found that a large amount of variation in gene expression correlated with the diagnostic categories (Fig. 4A and S9). However, no separate clusters were observed for typical versus atypical MEITL, nor for EATL in patients with or without a history of CeD.

By differential expression analysis (Table S4 and Fig. 4B), the most upregulated gene in EATL was *TNFRSF8* encoding CD30. Accordingly, CD30 expression was characteristic of EATL, usually with a moderate/strong staining intensity in >50% tumor cells (Table 1). Interestingly, there were several genes related to immune functions over-expressed in EATL. These included genes coding for *CD274*/PD-L1, *IDO1* (indoleamine-2,3 dioxygenase 1) which contributes to tumor immunosuppression by enzymatically degrading tryptophan [29], *CXCL13*, *CIITA*, *TGM2* (transglutaminase 2) which is an important enzyme and autoantigen in CeD [30], the vitamin D receptor and *CYP27B1*, an enzyme which catalyzes the production of vitamin D3, a factor promoting innate immunity and modulating adaptative immunity [31, 32]. Immunohistochemistry confirmed that EATLs contained a significantly higher proportion of CXCL13-positive cells than MEITL (4.4% versus 0.08%; *p* = 0.006). This was mostly related to microenvironment cells, but in addition, in four cases, CXCL13 expression was detected in large, atypical cells consistent with neoplastic cells (Fig. 4C). As expected, *NCAM1* encoding CD56 was expressed at higher levels in MEITL than in EATL. Other genes upregulated in MEITL included those coding for *MYBL1* transcription factor, the *PTPN4* tyrosine phosphatase, *PTGER2*, which is a receptor of prostaglandin E2, and the *KLFR2* receptor involved in natural killer (NK) cell activity.

Gene enrichment analysis (GSEA) using the hallmark signatures of cancer identified pathways related to cytokine signaling and inflammatory response over-expressed in EATLs (IL6_JAK_STAT3 and IL2_STAT5 signaling, IFN_alpha_response and INF_gamma_response, TNF_alpha signaling and Inflammatory_response pathway) (Fig. 4D). Differential TP53 pathway representation was in accordance with more frequent *TP53* loss-of-function mutations in MEITL [33, 34]. The enriched pathways in MEITL were associated with proliferation (E2F targets and G2M checkpoint).

Comparison of typical and atypical MEITL transcriptomes revealed 200 differentially expressed genes (Table S5). Signatures enriched in the atypical group (Fig. S10A) included five signatures related to proliferation (MYC_target v1 and v2, E2F_target, G2M_checkpoint, and mitotic_spindle). This is in keeping with the correlation with MYC expression [7] and a significantly higher proportion of cycling cells (Ki67 > 50%) in atypical (19/21, 90%) versus typical (19/30, 63%) MEITL (p = 0.048) (Fig. 1A). In EATL, there were only 28 genes differentially expressed according to history of CeD (Table S6). The hallmark oxydative_phosphorylation signature was the only signature enriched in tumors from patients with de novo EATL (Fig. S10B).

Unsupervised analysis of the miRNA expression did not separate MEITLs from EATLs, but differential expression analysis identified 593 miRNAs (Table S7), mostly over-expressed in MEITL (Fig. 4E, F). Among those, the miR-106a-363 cluster was the most over-expressed (Fold Change FC [12–94]), along with some members of two paralogous clusters (miR-17-92 and miR-106b-25). Integration of miRNAs with RNA-seq data (Fig. S11A), suggested that the highest potential impact on mRNA expression was related to several miR-105/107 family members expressed at higher levels in MEITL (Fig. S11B). Functionally, the target genes of the most impactful miRNAs were related to inhibition of PI3K-AKT-mTOR, cytokine and toll-like receptors signaling pathways, and upregulation of cell cycle and epithelial to mesenchymal transition pathways in MEITL (Fig. S11B).

### Deconvolution analyses of RNAseq and methylation profiles

**Fig. 5:**
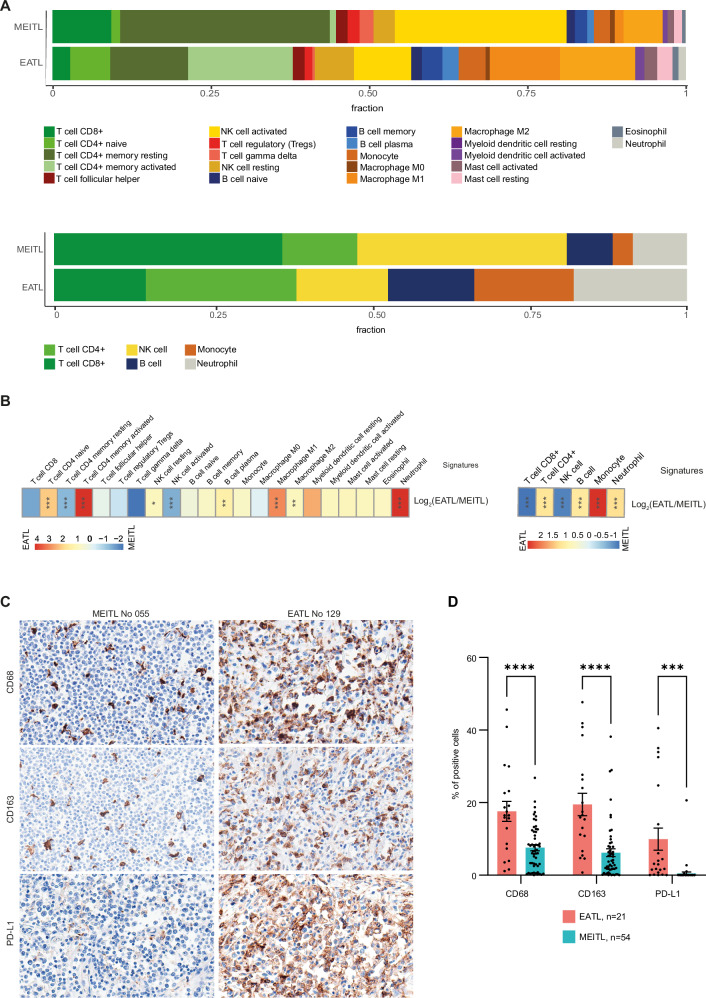
Deconvolution of RNA and methylome profiles. **A** Barplots showing the means of cell-type fractions observed by deconvolution analysis in 48 MEITLs and 27 EATLs based on gene expression (upper part) and in 39 MEITLs and 26 EATLs based on methylation profiles (lower part). Cell type composition was calculated for each sample based on methylation values using the *FlowSorted.Blood.EPIC* R package. **B** Heatmaps representations of the differences in proportions of immune cell subtypes between EATL and MEITL for deconvolution analysis of RNA sequencing (left) and global DNA methylation (right) data expressed in log_2_(mean (EATL)/mean (MEITL)), *, *p* < 0.05; **, *p* < 0.01 and ***, *p* < 0.001. **C** Immunostainings for macrophage markers (CD68, CD163) and PD-L1 in one MEITL (left) and one EATL (right). **D** Boxplots showing the comparisons of CD68, CD163 and PD-L1 percentages of positive cells between EATL (*n* = 21) and MEITL (*n* = 54) samples. ***, *p* < 0.001 and ****, *p* < 0.0001. Differences between groups were assessed by two-way ANOVA with Sidak’s multiple comparisons test.

Cellular deconvolution based on the gene expression and DNA methylation data, generated similar results (Fig. 5A, B), with quantitative and qualitative differences between EATL and MEITL. Remarkably, the proportion of cells with signatures corresponding to T- and NK-cell lineages was almost identical according to the two methods and represented a significantly higher proportion of the total tumor cell content in MEITL than in EATL, (72.5% versus 49.5%, *p* < 0.0001), reflecting a higher neoplastic cell density. Among T/NK signatures deconvoluted by gene expression, EATL tissues were enriched in T-cell CD4 naïve (*p* < 0.001) or memory activated (*p* < 0.001) and NK resting signatures (*p* < 0.05), while MEITLs were enriched in T-cell CD4 memory resting (*p* < 0.001) and NK activated signatures (*p* < 0.001), and showed a trend for enrichment in T.cell.ɣδ and T.cell.CD8 signatures (*p* = 0.08) (Fig. 5A). These findings reflect the cell derivation and immunophenotype of the neoplastic cells (Table 1). Regarding the composition of the microenvironment, overall more abundant in EATL than in MEITL, EATLs were specifically enriched in B cells, M1 (11% versus 1%; *p* < 0.001) and M2 (12% versus 6%; *p* < 0.01) macrophages and neutrophils (Fig. 5A, B, and Fig. S12).

Immunohistochemistry confirmed a higher density of CD68+ cells in EATLs than in MEITLs (17.6% versus 7.6%, *p* < 0.0001). Specifically, EATLs also comprised higher proportions of cells positive for CD163 (19.5% versus 6.2%, *p* < 0.0001) and PD-L1 (10% versus 0.5%, *p* < 0.001) associated with M2 macrophages (Fig. 5C, D).

### Distinctive methylation profiles in MEITL and EATL impacting gene expression

**Fig. 6:**
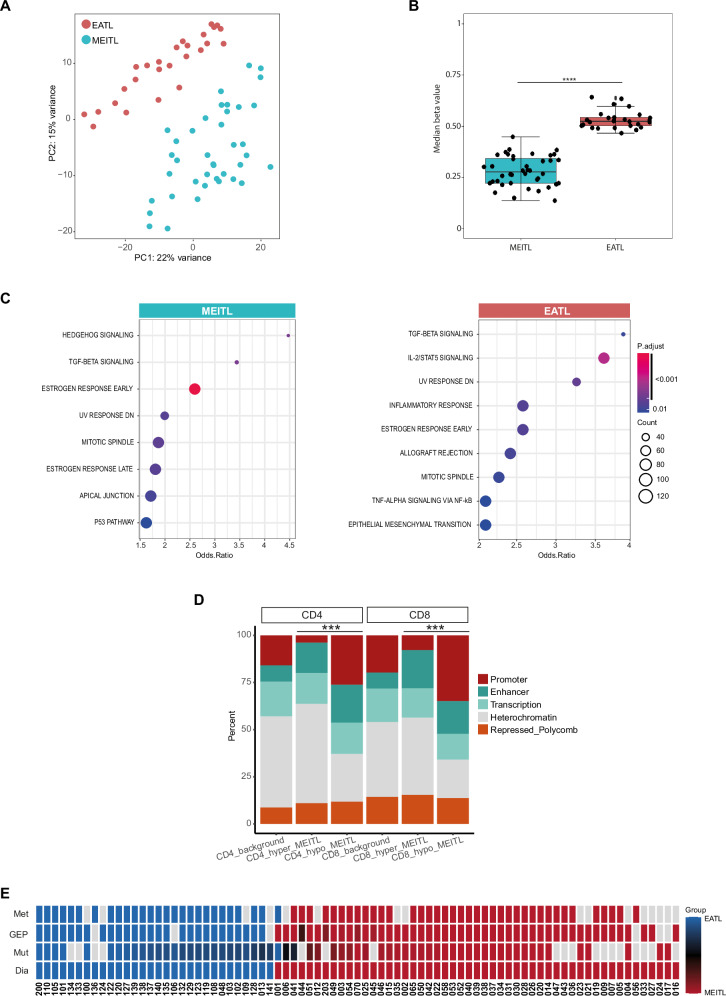
Methylome profile and consensus clustering on multiple omics. **A** Principal component analysis (PCA) of methylation profiles of 39 MEITLs and 26 EATLs. PCA displays the cases based on the 10’000 most variable CpGs, **B** Boxplot comparing the median methylation per sample for differentially methylated CpGs between MEITL and EATL. ****p < 0.0001. **C** Gene Enrichment Analysis using hallmark gene sets from the Molecular Signatures Database (MSigDB). The plot shows the signatures that were found significantly differentially enriched between MEITL and EATL with an adjusted *p* value ≤ 0.05 (corrected for multiple testing using Benjamini and Hochberg). Odds Ratio indicates the magnitude of the effect. **D** Distribution of differentially methylated CpGs between MEITL and EATL within chromatin states defined in CD4 and CD8 positive T cells. The distribution of all CpGs on the EPIC array is shown as control (background). Chi-squared test was performed to test if there is an enrichment of promoter CpGs within CpGs hypomethylated in MEITL. ***p < 0.001. **E** Consensus partitioning of mutations (Mut), gene expression (GEP) and methylome (Met) profiles was performed on a cohort of 51 MEITL and 30 EATL tumors. In the heatmap, the percentage of membership across all individual partitions is expressed as a color code, with blue associated with EATLs group and red with MEITLs. Samples with a weaker association with either group were colored in black. The last row represents the pathology diagnosis (Dia). Grey tile: data not available.

Unsupervised analysis of genome-wide DNA methylation profiles separated MEITL and EATL cases (Fig. 6A and Fig. S13). Considering differentially methylated loci, EATLs had a higher number of hypermethylated CpGs than MEITLs (Table S6, Fig. 6B). Pathway enrichment analysis of genes associated with differentially methylated CpGs showed enrichment of IL-2/STAT5 signaling in differentially hypomethylated CpGs in EATL (Fig. 6C). Mapping differentially methylated CpGs to chromatin states in CD4+ or CD8 + T-cells revealed CpGs hypomethylated in MEITL compared to EATL to be significantly enriched in promoter regions. (Chi-squared test, *p*-value < 0.001) (Fig. 6D). In conclusion, DNA methylation can be used to differentiate MEITL and EATL, and differences between both groups might affect gene expression via differential promoter methylation.

### Unsupervised consensus clustering consistently separates EATL from MEITL

Finally, we investigated the concordance between the assignment of the cases to the diagnoses based on the different OMICs levels by performing unsupervised consensus clustering on each of them. Except for the miRNA profile, the other three omics levels, namely mutational landscape, DNA methylome and gene expression, neatly split the population into two groups, which largely overlapped with tumor diagnosis (Fig. 6E), confirming distinct molecular profiles characterizing the two entities.

## Discussion

The current study provides an in-depth analysis of the clinicopathological features, genomic alterations, and molecular profiles of EATL and MEITL, in one of the largest mixed cohorts of intestinal T-cell lymphomas to date. Our findings underscore the marked differences in their oncogenic pathways and molecular characteristics, exemplified by the unsupervised analysis performed on different omics, which clearly segregated EATL from MEITL tumors.

The mutational landscapes comprised alterations in common key pathways, including epigenetics and JAK-STAT, but showed different patterns of affected genes, implying different oncogenic drivers. Most remarkably, the loss-of-function mutations in *SETD2* previously reported as the most recurrent alteration in MEITL[7, 13, 19] were not found in any of the EATL cases. Consistently, in the study by Cording et al. [19], none of the 19 EATL or 74 RCeD or CeD analyzed had *SETD2* alterations. Collectively, these findings clear the ambiguity that has persisted with the report of Moffitt AB et al. [14] that type I and type II EATL are characterized by the loss of function of SETD2. The current study establishes the specificity of *SETD2* mutations for MEITL in the context of the differential diagnosis with EATL. Instead, EATLs harbored frequent mutations in *TET2*, *ARID1A*, and *KMT2D*, which were rarely altered in MEITLs. Interestingly, these genes reportedly induce a hypermethylation phenotype [35–37], which is in line with the higher CpG methylation found in EATL compared to MEITL. The characteristic landscapes of mutations in genes governing epigenetic biology in EATL and MEITL suggest potential vulnerability to epigenetically targeted drugs such as histone deacetylase (HDAC) inhibitors or DNA hypomethylating agents [38]. More specifically, patients with MEITL might benefit from WEE1 inhibitors, which have a synthetic lethal effect in vivo on SETD2-deficient tumors in experimental conditions, although only limited clinical activity was demonstrated in patients with solid cancers [39].

A novel finding in MEITL was recurrent missense mutations in histone genes *H3-3A* and *H3-3B*, substituting arginine 2 of histone H3.3 with a cysteine or histidine or glutamine (p.Arg2Cys/His/Gln). The variants, therefore, disrupt the H3R2me2 mark, which antagonizes H3K4me3 at promoters while facilitating enhancer activity [40]. Interestingly, other rare tumors are associated with point mutations at other key amino acid residues of H3-3A/B [41, 42]. It was recently reported that the H3.3 G34 oncohistones associated with giant cell tumor of bone and pediatric gliomas selectively promote PRC2 activity by blocking SETD2-mediated H3K36 methylation [43, 44]. The co-alteration of *SETD2* and *H3-3A/B* in about a quarter of MEITL patients warrants further research into their interplay and highlights histone H3 mark deregulation as a central mechanism in MEITL.

Another notable discovery was the high frequency of HLA Class I genes and/or *B2M* alterations and associated loss of HLA-I molecules in EATL. Loss of MHC I antigen presentation is a frequent occurrence and a mechanism of immune evasion in many cancer types [45]. Yet, this pathway is underexplored in T-cell lymphomas, with limited available information. Genomic disruption of HLA class I genes has been reported in a small fraction of nodal peripheral T-cell lymphomas of follicular helper T-cell derivation and not otherwise specified [46, 47], and in 40% of patients with advanced cutaneous T-cell lymphoma [48]. In the latter study, the genetic events rarely affected total HLA-I protein but were associated with worse survival. In our cohort of EATL patients, there was no difference in overall survival according to the status of HLA class I genes (Fig. S6). However, these mutations may deserve consideration in patients eligible for salvage allogeneic transplantation or immunotherapy, as they may influence the response to therapy [49].

Alterations in the JAK-STAT pathway were overall prevalent in about 90% of the cases (35/37 MEITLs and 23/26 EATLs), suggesting potential sensitivity to a range of clinically available JAK- or STAT-inhibitors [50–53], and revealed key differences in immune signaling. Mutations in MEITL occurred in *JAK3* or *STAT5B*, while *STAT3* or *JAK1* were the main genes involved in EATL. The findings in EATL confirm and expand reports from smaller series [13, 14, 18, 19]. STAT3 and STAT5 are central to cancer-related inflammation and immunity [54, 55]. Moreover, in the evolution of CeD, inflammatory cytokines play a pivotal role in the neoplastic transformation of aberrant intraepithelial lymphocytes (IELs) [54, 56]. EATL is characterized by strong inflammatory activity, with enriched gene sets like interferon response and IL-6/JAK/STAT3 signaling and overexpressed inflammation-related genes (e.g., PD-L1, *CXCL13*), suggesting STAT3-driven tumor-promoting inflammation [29, 57–61]. Surprisingly, in consideration of the frequent *STAT5B* and *JAK3* mutations in MEITL, the IL2_STAT5_SIGNALING signature was enriched in EATL, perhaps as a result of increased cytokine secretion and signaling in the inflammatory environment of EATL.

The abundant microenvironment of EATL included both pro-inflammatory M1 and immunosuppressive [62, 63] M2 macrophage signatures [64–66] at higher levels than MEITL. This may reflect a dual-phase immune response, with M1 macrophages driving early tumor-promoting inflammation and M2 macrophages fostering immune evasion and tumor progression [62, 63]. Integrated with the frequent loss of HLA class I and antigen presentation, the co-existence of two opposing macrophage phenotypes in EATL may help explain the paradox of strong inflammatory signaling co-occurring with immunosuppression. Together, EATL appears driven by STAT3-mediated inflammation and immune evasion, while MEITL is a cytotoxic lymphoma with high expression of innate-like T/NK-cell genes (e.g., *STAT4, NCAM1*) and an “immune-cold” profile with low inflammation.

Considering the role of SETD2 in preserving genome integrity, its disruption likely contributes to the high level of chromosomal instability observed in MEITL, reflecting underlying defects in DNA repair and mitotic control. Indeed, MEITL displayed transcriptomic enrichment of E2F target genes and mitotic spindle signatures, suggestive of higher proliferation compared to EATL. *TP53* inactivation, prevalent in MEITL and rare in EATL, likely cooperates with *SETD2* alterations to exacerbate genomic instability and override DNA damage checkpoints. Together, these features support a model in which MEITL pathogenesis is driven by epigenetic deregulation and chromosomal instability, contrasting with the inflammation-associated oncogenesis of EATL.

In conclusion, the distinct genetic, epigenetic, and expression footprints and immunological landscapes of EATL and MEITL established by this study expand disease-defining features, have diagnostic implications, and provide a rationale for targeted therapies.

## Supplementary information

Supplementary material, methods and informations

Supplementary tables S1 to S8

## Data Availability

Whole-exome sequencing and RNA sequencing data have been submitted to the European Genome-phenome Archive (EGA). Study accession numbers: EGAS50000001125 and EGAS50000001126.
