# Correction: Divergent molecular pathways drive monomorphic epitheliotropic and enteropathy-associated intestinal T-cell lymphoma

**DOI:** 10.1038/s41375-025-02808-y

**Published:** 2025-11-20

**Authors:** David Vallois, Edoardo Missiaglia, Luis Veloza, Anja Fischer, Doriane Cavalieri, Vimel Rattina, Bettina Bisig, Vincent Roh, Laura Wiehle, Rita Sarkis, Emmanuel Bachy, Christophe Bonnet, Julie Bruneau, Anne Cairoli, Roland De Wind, Fanny Drieux, Romain Dubois, Jean-François Emile, Virginie Fataccioli, Kamel Laribi, Albane Ledoux-Pilon, François Lemonnier, Francisco Llamas-Gutierrez, Pierre Morel, Marie Parrens, Elsa Poullot, Leticia Quintanilla-Martinez, Jeremy Sandrini, Joan Somja, Luc Xerri, Olivier Tournilhac, Philippe Gaulard, Reiner Siebert, Laurence de Leval

**Affiliations:** 1 Institute of Pathology, Lausanne University Hospital and University of Lausanne, Lausanne, Switzerland; 2 Institute of Human Genetics, Ulm University and Ulm University Medical Center, Ulm, Germany; 3 Department of Hematology, University Hospital of Lille, Lille, France; 4 Translational Data Science Facility, AGORA Cancer Research Center, Swiss Institute of Bioinformatics, Lausanne, Switzerland; 5 Department of Hematology, Centre Hospitalier Lyon Sud and Inserm, U1111 Pierre Bénite, France; 6 Department of Hematology, University Hospital Center of Sart Tilmanand and Liège University, Liège, Belgium; 7 Department of Pathology, Necker Hospital for Sick Children, AP-HP, Paris-Cité University, Paris, France; 8 Service of Hematology, Department of Oncology, Lausanne University Hospital and Lausanne University, Lausanne, Switzerland; 9 Department of Pathology, Institute Jules Bordet, Bruxelles, Belgium; 10 Centre Henri Becquerel, Service of Anatomical and Cytological Pathology, Centre Henri Becquerel, Rouen, France; 11 Department of Pathology, University Hospital of Lille, Lille, France; 12 Department of Pathology, Ambroise Paré Hospital - Université Saint Quentin en Yvelines, Paris, France; 13 Department of Pathology, AP-HP, Henri Mondor Hospital, F-94010 Créteil, France; 14 University Paris Est Créteil, INSERM, IMRB, Créteil, France; 15 Department of Hematology, Hospital Centre Le Mans, Le Mans, France; 16 Department of Pathology, University Hospital of Clermont-Ferrand, Clermont-Ferrand, France; 17 AP-HP, Henri Mondor Hospital, Lymphoid malignancies unit, Créteil, France; 18 Department of Pathology, University Centre Hospital, Rennes, France; 19 Department of Hematology, Hospital of Lens, Lens, France and Department of Hematology, University Hospital of Amiens, Amiens, France; 20 Department of Pathology, Bordeaux University Hospital, Bordeaux University, Bordeaux, France; 21 Institute of Pathology, University Hospital Tübingen, Eberhard Karls University of Tübingen, Tübingen, Germany; 22 Department of Pathology, Le Mans Hospital Center, Le Mans, France; 23 Institut Paoli-Calmettes, CRCM and Aix-Marseille University, Marseille, France; 24 Department of Hematology, University Hospital of Clermont-Ferrand, Clermont-Ferrand, France; 25 German Center for Child and Adolescent Health (DZKJ), partner site Ulm, Ulm, Germany

**Keywords:** Oncogenesis, Cancer genomics, Cancer epigenetics, T-cell lymphoma

Correction to: *Leukemia* 10.1038/s41375-025-02777-2, published online 07 October 2025

In the originally published version of this article, same minor errors were present: (1) in Table 1, only the headers should appear in bold font, as now corrected; (2) in Figure 1B, two typographical errors occurred in panels c and d, where the correct legends are MEITL No 030 and MEITL No 039 instead of EATL No 030 and EATL No 039; (3) in Figure 3B, the MEITL CNV profile was mistakenly replaced by a duplication of the EATL profile. The correct MEITL profile has been now inserted; (4) in Figure 4F, the miRNA “577” label appeared in green font instead of black. All errors have been corrected in the revised figures and table provided in the updated version.

Incorrect figure 1
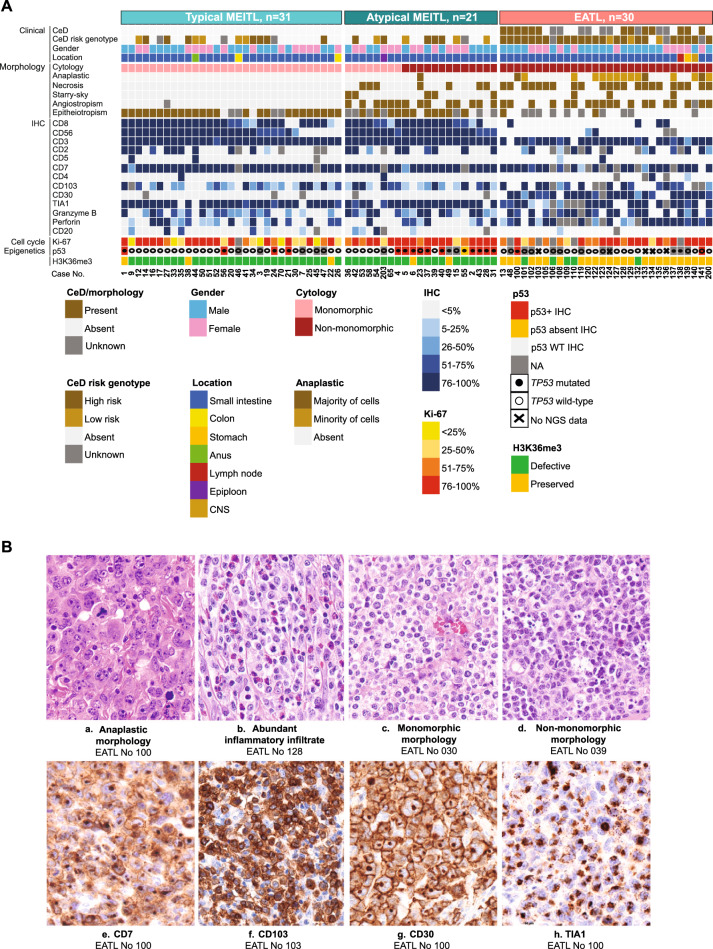


Corrected figure 1
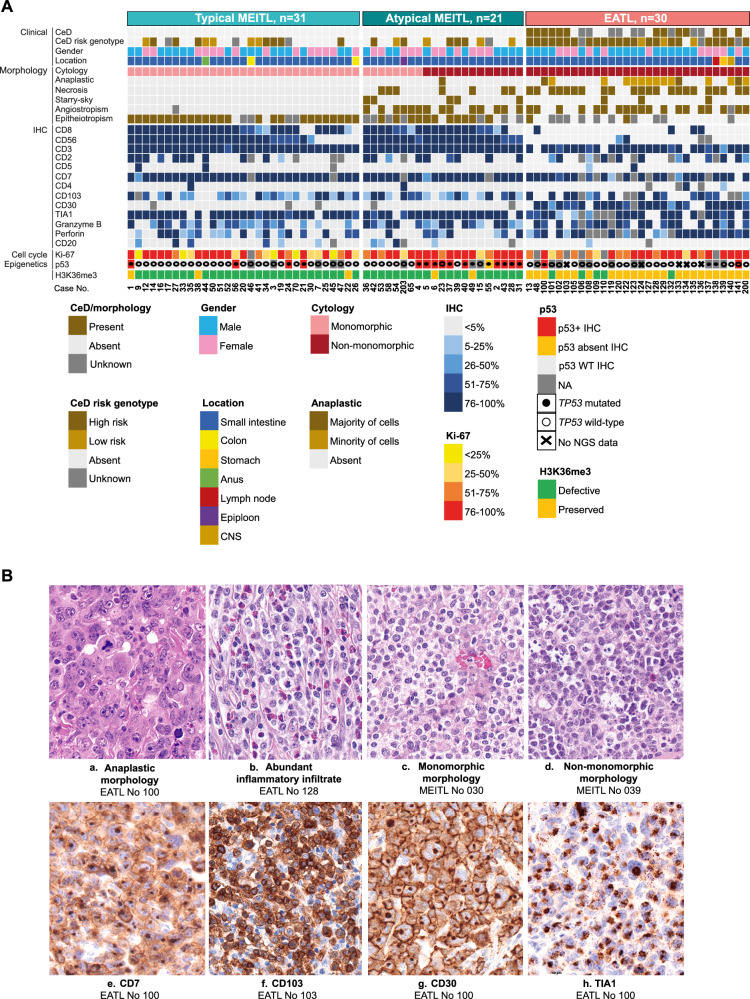


Incorrect figure 3
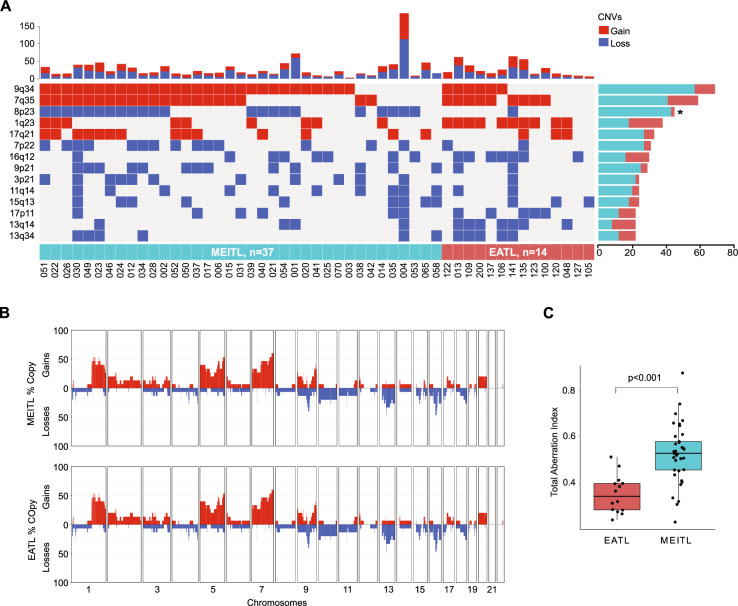


Corrected figure 3
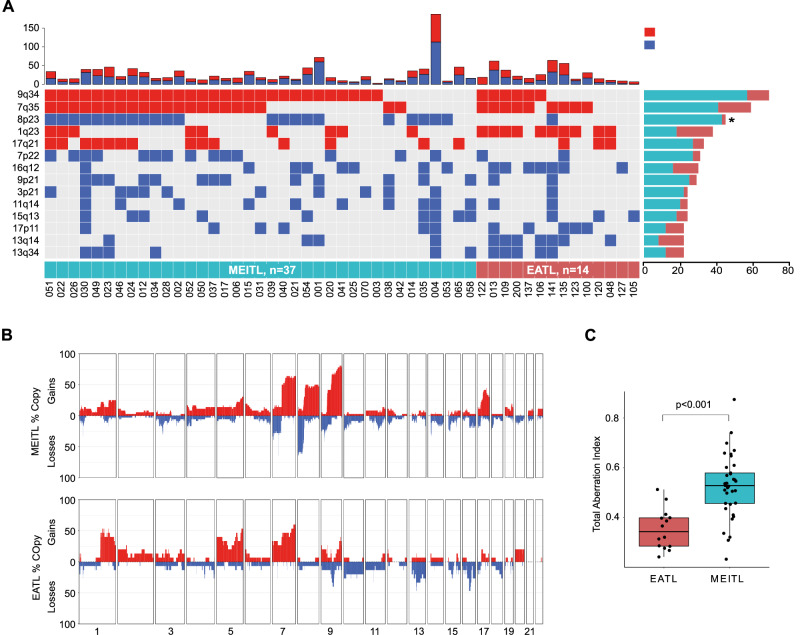


Incorrect figure 4
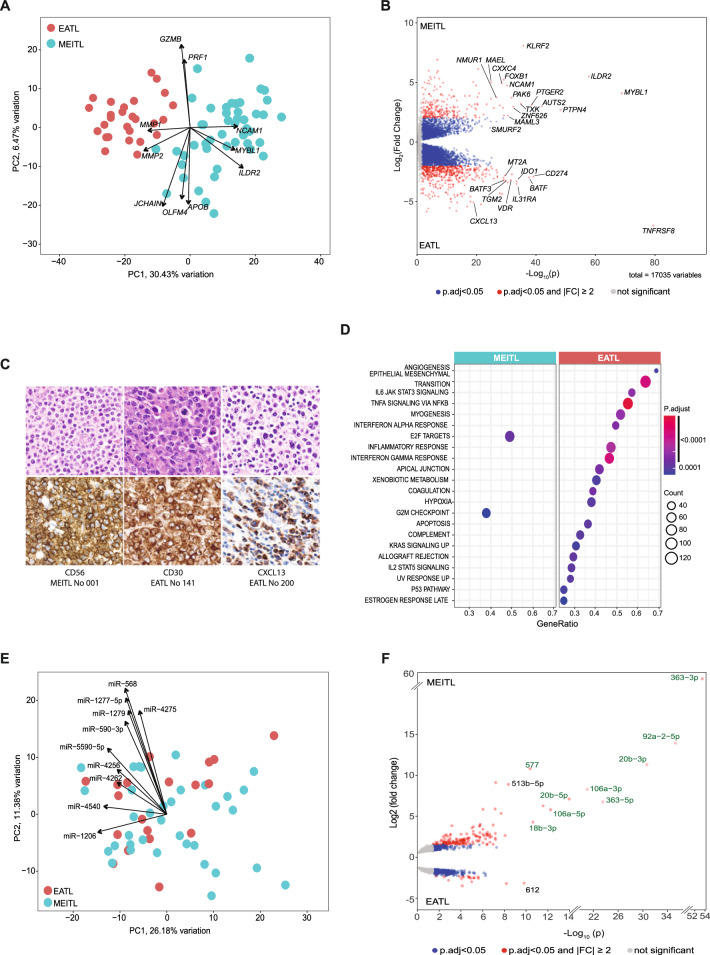


Corrected figure 4
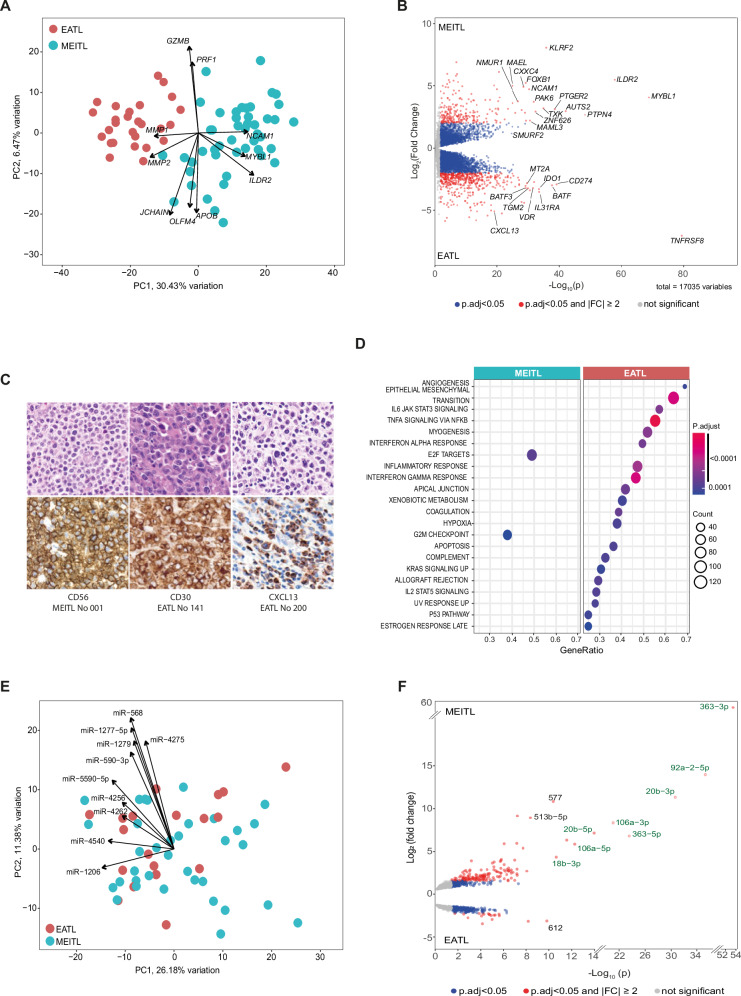

**Table:** 

	MEITL (*n* = 52)	EATL (*n* = 30)	*p* value
**Clinical features**			
Reported celiac disease	0/52 (0%)	13/30 (43%)	**<0.005**
Age, median, years (range, median)	67 (29-91)	64 (34-86)	0.37
Gender (male/female)	27:25:00	19:11	0.2
Acute event at presentation	38/44 (86%)	12/18 (67%)	0.09
Bowel perforation	33/44 (75%)	8/18 (44%)	**0.037**
Bowel obstruction	12/44 (27%)	6/18 (33%)	0.76
Lugano stage			
Stage I/II	27/44 (61%)	8/13 (61%)	1
Stage III/IV	17/44 (39%)	5/13 (39%)	
PS			
0-1	18/40 (45%)	4/8 (50%)	1
≥2	22/40 (55%)	4/8 (50%)	
**Morphology**			
Typical	31/52 (60%)		
Atypical	21/52 (40%)		
Pleomorphic	13/52 (25%)	30/30 (100%)	**<0.005**
Anaplastic	1/52 (2%)	15/30 (50%)	**<0.005**
Necrosis	7/52 (13%)	22/30 (73%)	**<0.005**
Starry-sky	5/52 (10%)	3/30 (10%)	1
Angiotropism	14/51 (27%)	19/29 (65%)	**<0.005**
Epitheliotropism	36/41 (88%)	2/20 (10%)	**<0.005**
Moderate/abundant inflammation	3/52 (6%)	20/30 (67%)	
**Immunophenotype**			
CD8	47/52 (90%)	4/30 (13%)	**<0.005**
CD56	46/52 (88%)	2/28 (7%)	**<0.005**
CD3	52/52 (100%)	27/30 (90%)	**0.046**
CD2	20/47 (42%)	12/24 (50%)	0.619
CD5	2/51 (4%)	2/30 (7%)	0.624
CD7	48/48 (100%)	22/23 (96%)	0.324
CD4	2/52 (4%)	2/27 (7%)	0.603
CD103	40/51 (78%)	15/25 (60%)	0.108
CD30	0/47 (0%)	25/29 (86%)	**<0.005**
TIA1	47/52 (90%)	20/25 (80%)	0.108
Granzyme B	37/52 (71%)	19/28 (68%)	0.596
Perforin	27/52 (52%)	22/29 (76%)	**<0.005**
CD20	9/49 (18%)	1/29 (3%)	**0.046**
CD79a	3/41 (7%)	0/10 (0%)	0.567
**TCR**			
TCRβ	17/50 (34%)	8/28 (29%)	0.472
TCRγδ	26/50 (52%)	8/27 (30%)	**0.05**
TCRαβ-TCRγδ+	22/49 (44%)	4/26 (15%)	**0.012**
TCRαβ+TCRγδ-	12/49 (24%)	4/26 (15%)	0.391
TCRαβ+TCRγδ+	4/49 (8%)	3/26 (11%)	0.685
TCRαβ-TCRγδ-	11/49 (22%)	15/26 (58%)	**0.004**

The original article has been corrected.

