# Supplementary material for: Divergent molecular pathways drive monomorphic epitheliotropic and enteropathy-associated intestinal T-cell lymphoma
Source: Leukemia. 2025 Oct 7;40(1):106–19. doi: 10.1038/s41375-025-02777-2 (PMC12789032; doi:10.1038/s41375-025-02777-2)
Supplement: Supplementary file 1 — Supplementary material, methods and informations [file 41375_2025_2777_MOESM1_ESM.pdf]

Divergent molecular pathways drive monomorphic epitheliotropic and enteropathy-associated intestinal T-cell lymphoma

David Vallois, Edoardo Missiaglia et al.

Supplemental material and methods

Cases studied

Eighty-two patients diagnosed with EATL or MEITL between 2000 and 2022 were collected from several institutions in Switzerland, France, Belgium and Germany and through the T-cell lymphoma working group of the LYSA (Lymphoma Study Association). 52 MEITLs and 6 EATLs were included in previous studies.(1, 2) Demographic and clinical data were obtained from the patients' files by the treating physicians or from the histopathological reports.

Histology, immunohistochemistry and fluorescence *in situ* hybridization

Diagnostic slides were reviewed, and representative formalin-fixed paraffin-embedded (FFPE) tumor tissue block(s) were collected. The cases were centrally reviewed (LV, LdL, PG) according to the revised 4th WHO classification criteria.(3) Additional studies (immunostainings, EBER in situ hybridization for EBV detection and fluorescence in situ hybridization (FISH) assays) to complement diagnostic evaluation were performed, interpreted and recorded along with histopathological features in a coded dataset as previously described.[10]

Immunohistochemistry validations

In order to validate some findings derived from mutation or gene expression analysis at the protein level, immunohistochemistry (IHC) assays with CD68, CD163, PD-L1, CXCL13 and HLA-1 antibodies (table below) were performed on previously designed tissue microarray (TMA). These sampled 54 MEITL and 21 EATL cases, including 34 MEITL and 15 EATL cases from the current study cohort. The TMA blocks were constructed with two cores of 1 mm diameter per case. TMA IHC images were annotated per case using QuPath 0.5.1, an open-source digital pathology software.(4) Color contrast thresholds were applied to Harris hematoxylin, DAB, residual, and optical density sum channels to optimize marker detection. Positive cell detection was performed in QuPath with parameters set to detect cells based on optical density sum, a pixel size of 0.26 µm, and cell expansion of 2.5 µm. Scoring was compartment-specific, focusing on the nucleus DAB optical density mean for the markers CD68, CD163, PD-L1 and CXCL13 to quantify expression levels accurately. Results were

exported per annotation as a .csv file. HLA-1 immunostaining was additionally performed in 15 EATL cases. Thus, HLA staining was contributory in 29 EATL and 34 MEITL cases. HLA staining was evaluated semi-quantitatively (absent/reduced expression: 50% or less positive tumor cells; preserved expression > 50% positive tumors cells).

Antibody	Clone	Source	Dilution
CD68	PG-M1	DakoCytomation, Agilent Technologies, Santa Clara, CA	1 : 50
CD163	10D6	Novocastra, Newcastle, UK	1 : 200
PD-L1	SP263	Ventana Medical Systems, Tucson, AZ	RTU
CXCL13	Polyclonal	Invitrogen, Waltham, MA	1 : 500
HLA-1	EMR8-5	Abcam, Cambridge, UK	1 : 2000

Genomic DNA and RNA preparation

4 µm thick FFPE sections were manually microdissected to pick up tumoral or non-tumoral tissues. Genomic DNA (gDNA) and RNA were extracted using the Maxwell 16 tissue DNA purification kit and the Maxwell 16 LEV RNA FFPE kit respectively, with a Maxwell machine (Promega, Madison, Wisconsin, USA). gDNA and RNA were quantified using a Qubit machine (ThermoFisher, Waltham, Massassuchetts, USA) and analyzed with a fragment analyzer (Agilent, Santa Clara, California, USA).

Whole exome sequencing (WES)

Fourteen MEITLs previously reported had been sequenced using the SureSelectXT Target Enrichment System (Agilent Technologies, Santa Clara, CA, USA).(1) Twenty-six EATLs and 23 additional cases of MEITLs (among which 5 had only tumoral tissue available) were sequenced using the xgen research panels v1.0 and v2.0 from IDT DNA (Newark, New Jersey, USA). Briefly, 500 ng of paired tumoral and normal DNA extracted from micro-dissected FFPE tissues were sheared with Covaris S220. Libraries were prepared with the KAPA HyperPlus kit (Roche, Pleasanton, CA) combined to the xGen research panel v1.0 and v2.0 and UMIs (Unique Molecular Indexes) from IDT DNA and paired-end sequenced on a HiSeq 4000 from Illumina (San Diego, CA). Demultiplexing, alignment, duplicate removal, variant calls and filtering and visual validation of all variants were performed as previously described.(2) On average, around 80 million pair-end reads per sample were sequenced reaching a mean coverage of 201X (range 83-536) for tumors and 177X (range 20-380) for normals.

WES derived analyses

Tumor Mutational Burden (TMB)

TMB was computed by dividing the number of non-synonymous mutations (missense, nonsense, frameshift and inframe-indel) with the size of the exome panel included in the coding sequence of the human genome (hg19 assembly). Specifically, 33.2Mbs for the IDT panel and 33.9Mbs for the Agilent v5 panel.

Copy Number Variations analysis

Somatic copy number variants were detected using GATK CNV v4.1 pipeline starting from the alignment files of samples with an estimated tumor content higher than 40% by morphological evaluation (14 EATL and 37 MEITL). Data standardization and denoising were performed using a Panel Of Normal (PON) generated from 54 normal samples. Common minimal regions (CMR) were identified using Genomic Identification of Significant Targets In Cancer (GISTIC v2.0) with a confidence level of 0.99 (-conf), 15 as smallest number of markers for a segment to do not be joined to the neighboring segment (-js) and 0.1 as significance threshold for q-values (-qvt). Chromosomal instability scores were computed using the CINmetrics package (v.0.1.0) importing the segmented data in R software.

RNA sequencing (RNA seq)

Libraries from 48 MEITLs and 27 EATLs were prepared starting from 1 μg of total RNA using the TruSeq Stranded total RNA Gold kit from Illumina (San Diego, California, USA) and were sequenced in three batches on a HiSeq 4000 machine from Illumina with an average output of 63 million reads per sample (range 49-126). The raw fastq files were processed by fastQC (v0.11.9) and multiQC (v1.9) for quality assessment. Genes expressions were quantified by Salmon (v1.3.0.) tool through tximeta package (v1.20.1). Salmon index was built using GRCh38 genome and gencode v35 annotation files. Further quality assessments, including reads and biotype distributions and saturation, were performed using NOISeq (v2.38.0) R package.

RNAseq derived analyses

Differential expression analysis

Differential expression analysis was performed on protein coding genes by *DESeq2* (v1.42.0) and *edgeR* (v4.0.6) packages using a model including histological subtypes and batches and

applying a filter to remove low expressing genes (<10 counts in 5% of the samples). Genes were considered differential expressed between the two subtypes if they showed a fold-change above 2 and an adjusted (Benjamini-Hochberg method) p-value < 0.05.

Genes read counts were normalized using a variance stabilizing transformation in DESeq2 package, followed by batch effect removal by *limma* (v3.58.1) R package. Unsupervised hierarchical clustering was applied to the top 300 genes showing the higher interquartile range using Euclidian distance and complete-linkage clustering in the *pheatmap* (v1.0.12) R package. Moreover, for dimensionality reduction we applied principal component analysis (PCA) on top 2% most variant genes as well as uniform manifold approximation and projection (UMAP) with default configuration on the full gene set using the *PCAtools* (v.2.14) R package and with *umap* (v.0.2.10) R package, respectively.

Functional enrichment analysis was performed starting from the signed log2 transformed p-values obtained by the differential expression analysis applying the Gene Set Enrichment Analysis method in the *ClusterProfiler* (v4.17.0) package with the hallmark datasets obtained from the Human Molecular Signatures Database (MSigDB – *msigdbr* package v24.1.0).

HLA haplotypes determination

HLA haplotyping was performed using RNAseq data upon alignment to the HLA-I-II CDS (EBI database) with BWA-MEM (v.) followed by HLA prediction with *HLAminer* v.1.4. The prevalence of the HLA-DQB1*02 and the haplotypes associated with high risk of developing celiac disease, including HLA-DQ2.5 (HLA-DQA1*05/DQB1*02) and HLA-DQ8 (HLA-DQA1*03/DQB1*0302), were compared to those observed in the general European population using "The Allele Frequency Net Database" (https://www.allelefrequencies.net/) which gathers alleles, haplotypes and genotypes frequencies in worldwide population.

Deconvolution

Deconvolution of cell-type fractions was performed through *immunedeconv* (v2.2.2) package with a focus on CIBERSORT method with its LM22 gene matrix. The differences in the proportion of immune cell subtypes between EATL and MEITL were assessed by log2 foldchange, using the MEITL group as the reference. Significance was tested using two sided Mann–Whitney U test.

microRNA profiling

We used the HTG EdgeSeq miRNA Whole Transcriptome Assay workflow and the HTG EdgeSeq system (HTG Molecular Diagnostics, Tucson, Arizona, USA) to profile miRNAs expression in 19 EATLs and 35 MEITLs, starting from 25 mm^2 of tumor tissue isolated from FFPE tissue sections and processed following the manufacturer's guidelines. Libraries were sequenced on a MiSeq sequencer (Illumina). Data were analyzed using HTG reveal software. Differential expression was performed with *DESeq2* software (quantile normalization).

Genome-wide DNA methylation profiling

To measure DNA methylation, 500 ng of genomic DNA was bisulfite-converted using the EZ DNA Methylation kit (ZymoResearch) according to the manufacturer's instructions. DNA methylation profiles were generated using the Infinium MethylationEPIC BeadChip (Illumina). Raw idat files were normalized using the preprocess Illumina function without background correction from the *minfi* package within R (v.4.3.1). Subsequently, beta values were calculated representing the percentage of DNA methylation at a certain cytosine base. For downstream analysis, loci on gonosomes were excluded from further analysis. Based on variance between all samples, the 10,000 most variable CpGs were selected and displayed as unsupervised analysis. PCA was performed using the prcomp function and heatmaps were generated with the *pheatmap* package. Differentially methylated loci were calculated using the *limma* package. P values were adjusted using the Benjamini-Hochberg method and CpGs were filtered for FDR. Pathway enrichment analysis was performed using the *enrichR* package with the MSigDB_Hallmark_2020 database. Cell type composition was calculated using the *FlowSorted.Blood.EPIC* package. Tumor cell content was calculated using the *InfiniumPurify* package with normal lymphoid FFPE tissue as control.

Chromatin states from benign CD4+ (n = 4: C002TWH1, C002Q1H1, S007DDH2, S007G7H4) and CD8+ (n = 3: C002YMH1, C0066PH1, S00C2FH1) T cells were downloaded from the Blueprint project (https://ftp.ebi.ac.uk/pub/databases/blueprint/releases/20150128/homo_sapiens/secondary_analysis/Segmentation_of_ChIP-Seq_data/) and overlapped with CpGs on the Infinium MethylationEPIC BeadChip (background) and differentially methylated CpGs between MEITL and EATL (n = 10,233). Further, the 12 chromatin states were collapsed into 5 functional chromatin states [PMID: 28934481]. Merged values for CD4+ and CD8+ T cells are shown. Chi squared test was performed on absolute CpG counts to test enrichment of specific chromatin states.

Integrative analyses

WES-RNAseq integration

The levels of expression of all somatic mutations identified by WES were extracted from the RNAseq data. This was achieved using *bam-readcount* (v.0.8.0) and *Strelka* (v.2.9.10 with configureStrelkaGermlineWorkflow.py and --rna flag) on the RNAseq reads aligned using *STAR* aligner (v.2.7.9a14) on the hg19 reference genome. For each gene, we counted the number of variants which were present in the transcripts (at least once) or those for which only the wild-type sequence was detectable. In some samples no transcript was detected at the specific variant location. Fisher-exact test was employed to assess the significance of the difference when compared to the average number of variants observed across all. Benjamini-Hochberg procedure was applied for Multiple testing adjustment.

microRNA profiling and RNA seq integration

Genes found differentially expressed between EATL and MEITL were processed through *multiMiR* (v1.24.0) R package to find their miRNA interacting partners. We considered only miRNA target interactions (MTI) databases experimentally validated (mirecords, mirtarbase and tarbase), excluding interactions reported as "Weak", "negative" or "Non-Functional". The final list was crossed with that of the miRNAs found differentially expressed between the two entities. This produced two lists: genes found down-regulated known to be targeted by miRNA found up-regulated in EATL or MEITL, respectively. Each list was processed through *miRmapper* (v0.0.0.9000) R package to identify and rank the most impactful miRNAs within EATL or MEITL. Finally, mRNAs targets of those miRNAs were functionally annotated using *EnrichR* (v3.2) and *Cytoscape* (v3.8.2) softwares. The procedure is shown in Supplementary Figure S11A.

Unsupervised standard consensus partitioning

Normalized and transformed data from RNAseq, miRNA and methylome were analyzed using *cola* package (v.2.12.0) in R using the full spectrum of variance selection (SD, MAD, CV, ATC) and partition methods (hclust, mclust, k-means, skmeans, pam). Moreover, we used the following setting: k_max=4, gene sampling=80%, partition repeat=50 and top_genes= 300, 1000, 2000 (for RNAseq), top_genes= 200, 300, 500 (for miRNA) and top_genes= 5000 (for methylome). After the initial variant filtering, we started the partitioning procedure using 18253 genes (RNAseq), 1978 miRNAs (miRNA) and 20000 CpG sites (methylome).

For mutation profile, we created a binary matrix with each row representing genes, and each column a sample, where value 0 denoted a wildtype status and 1 if at least one mutation was detected in the specific sample. Starting from a set of genes found mutated in at least 6 samples, we performed consensus partitioning computing a dissimilarity matrix based on Manhattan distance and Ward.D2 as clustering method. The analysis was performed using the following settings: k_max=4, gene sampling=80%, partition repeat=50 and top_genes= 10, 20, 30, 40.

List of Supplemental Tables

Supplemental Table S1. List of the 7101 mutations (SNVs, small indels) found by WES in 37 MEITLs and 26 EATLs.

Supplemental Table S2. MutSig2CV results from the analyses of the whole cohort (n=63), MEITL only (n=37) and EATL only (n=26).

Supplemental Table S3. Proportion of single nucleotide variants observed by WES which were also detected in the transcripts of the same samples (24 EATLs and 35 MEITLs).

Supplemental Table S4. Differentially expressed genes between MEITL (n=48) and EATL (n=27) with p adjusted value <.05 and a Fold Change > 2.

Supplemental Table S5. Differentially expressed genes between typical (n=28) and atypical (n=20) MEITL with p adjusted value <.05 and a Fold Change > 2.

Supplemental Table S6. Differentially expressed genes between EATL in patients with CeD history (n=12) and EATL in patients without CeD history (n=8) with p adjusted value <.05 and a Fold Change > 2.

Supplemental Table S7. Differentially expressed miRNA between MEITL (n=35) and EATL (n=19) with p adjusted value <.05.

Supplemental Table S8. Differentially methylated loci (DMLs) in MEITL (n=39) vs EATL (n=26).

Legends to Supplemental Figures

Supplemental Figure S1. Flow chart of the study.

Venn Diagrams show the number of cases analyzed by individual molecular studies and combinations thereof.

Abbreviations: EATL: entreropathy-associatied T-cell lymphoma; FFPE: formalin-fixed paraffin embedded; MEITL: monomorphic epitheliotropic intestinal T-cell lymphoma; QC: quality control; TMA: tissue microarray; IHC: Immunohistochemistry

Supplemental Figure S2. HLA genotypes. (A) Distribution of alleles and haplotypes related to high and low risk of celiac disease development in 75 patients with EATL or MEITL. Differences were assessed by Fisher's exact test. *, p<0.05; ****, p<0.0001. There is no difference in these distributions between patients with EATL with CeD history vs without CeD history (p=0.53). **(B)** Frequencies of HLA-DQ2.5 and HLA-DQ8 haplotypes and HLA-DQB1*02 allele in the 75 patients of this cohort in comparison to the general European populations obtained from "The Allele Frequency Net Database". Differences were assessed by Fisher's exact test with multiple test comparison correction. ***, p<0.001.

Supplemental Figure S3. Overall survival of 69 patients with available follow-up. (A) Comparison of patients with EATL (n=24) or MEITL (n=45). **(B)** Patients with MEITL were further analyzed according to morphologic subtypes, tumor stage and performance status, **(C)** and those with EATL according to celiac disease status and cytomorphology. The Kaplan-Meier curves were compared by the log-rank test and P-values adjusted for multiple testing by the Benjamini-Hochberg procedure.

Supplemental Figure S4. Mutational landscapes. (A) Comparison of mutation frequencies in MEITL and EATL for genes related to epigenetics, JAK/STAT signaling and MAPK pathway. (B) Mapping of somatic mutations in *JAK1, STAT3, TP53, JAK3, STAT5B, H3-3A/B* and *SMCHD1* proteins. Protein domains are represented according to the UniProt database (http://www.uniprot.org). All somatic mutations identified by WES in the 26 EATLs and 37 MEITLs analyzed in this study are shown. Colored circles indicate mutations found in the same samples. Mutations are colored by the type of alteration. Thirteen of the 15 *STAT3* mutations

in EATL occurred at 5 amino-acids positions altering its C-terminal SH2 domain, and two mutations occurred at one amino-acid position in the all-alpha domain of the protein. Most of these mutations have been characterized as activating(5-7). Eleven of the 14 *JAK1* mutations in EATL in the C-terminal protein kinase 2 domain correspond to activating variation of the amino-acid G1097(5, 8). The distribution of the *JAK1* mutations found in 5 MEITLs was different, with 5/7 affecting the protein kinase 1 domain. Mutations in *H3-3A* and *H3-3B* are specific to MEITL, and all missense mutations affect the arginine in position 2 of the protein. *H3-3-A* harbored p.R2C and p.R2H in 7 and 1 cases respectively. *H3-3B* harbored p.R2Q mutation in 2 cases.

Supplemental Figure S5. Overall survival of the 24 patients with EATL and available follow-up according to mutated versus wild-type status of *STAT3, JAK1, TET2*, *ARID1A* and HLA class I genes. The Kaplan-Meier curves were compared by the log-rank test and P-values adjusted for multiple testing by the Benjamini-Hochberg procedure.

Supplemental Figure S6. Overall survival of the 45 patients with MEITL and available follow-up according to mutated versus wild-type status of *H3-3A/B, TP53, STAT5B* and *SMCHD1*. The Kaplan-Meier curves were compared by the log-rank test and P-values adjusted for multiple testing by the Benjamini-Hochberg procedure.

Supplemental Figure S7. Expression of mutated genes. MEITL (n=35) and EATL (n=24) samples for which both WES and RNAseq data were generated, are shown. Cases and genes are represented by columns and rows, respectively, ordered as in Figure 2. Overall, 51% of the non-synonymous single nucleotide variants (SNVs) detected by WES (2748/5396) were covered with at least one transcript read and 1484 (28%) with more than 10 reads, with no significant differences between oncogenes and suppressor genes or missense and nonsense variants. For each somatic mutation, RNA sequencing data were analyzed to assess the number of transcripts carrying the wildtype or mutant sequence. Squares indicate the presence of reads covering the mutation sites, with their sizes indicating the depth of coverage (Depth range) for the specific site, and colors indicating the presence (MUT) or not (WT) of the mutation and range of mutated transcripts. Circles denote the absence of reads covering the mutation sites. Violin plots on the right side show the overall levels of expression of each gene in MEITL and EATL separately. It is apparent that mutations in low expressing genes, such as *FAT3*, *LRP1B* and *CSMD3*, do not generate detectable transcripts. On the other side, driver genes, such as

SETD2, *STAT5B*, *JAK3*, *JAK1* and *STAT3*, consistently show overall high level of expression, with presence of the mutated transcripts.

Supplemental Figure S8. Recurrent Copy Number Variations (CNVs) in 14 EATLs and 37 MEITLs. GISTIC 2.0-defined recurrent copy number focal regions (peak limits) affected by deletions (blue, left) and gains (red, right) are visualized as mirror GISTIC plots in MEITL and EATL. Chromosomes are on the vertical axis. G score is on the x-axis. The G-score quantifies the amplitude and frequency of CNVs across a cohort, integrating how often a genomic region is altered and how strongly it is gained or lost. Green line denotes the significance level at q-value 0.1. For significant CNVs, the corresponding cytoband/arm with selected genes of interest are indicated. Genes included in the peak limits are in green font while genes included in wide peaks are in black font. No significant recurrent CNV was found among EATLs.

Supplemental Figure S9. Unsupervised hierarchical clustering by expression levels of the top 400 most variable genes. Each line is an individual gene, and samples **(**48 MEITLs and 27 EATLs) are displayed as columns. They are segregated in two groups completely overlapping with the diagnostic categories. Subgroups according to morphology (typical and atypical MEITLs), or clinical history (EATL from patients with history of celiac disease versus others) were not individualized by their gene expression profiles with the unsupervised clustering.

Supplemental Figure S10. Genes Enrichement Analysis using hallmark gene sets from the Molecular Signatures Database (MSigDB) (https://www.gseamsigdb.org/gsea). The plots show the signatures which were found significantly differentially enriched between **(A)** typical vs atypical MEITL or **(B)** EATL without CeD vs with CeD with an adjusted p value ≤0.01 (corrected for multiple testing using Benjamini and Hochberg). The Gene Ratio represents the proportion of genes that are over-expressed within a specific pathway or signature, relative to the total number of genes associated with that pathway or signature.

Supplemental Figure S11. Identification of relevant miRNA-mRNA pairs (A) Method for microRNA profiling and RNA seq integration. For details, refer to the "*microRNA profiling and RNA seq integration*" paragraph from supplemental methods. DE: Differentially Expressed **(B)** Top 5 miRNAs most impactful on gene expression in MEITL and EATL. *Left panel.* Members of the miR-105/107 group are indicated in red. *Right panel*. Cytoscape and EnrichR

analysis of the mRNAs targeted by the most impactful miRNAs, using the hallmark_2020 genes set from MSigDB, showed the pathways impacted and their variations in MEITL and EATL. *Low*er panel. Venn diagrams showing the numbers of mRNAs targeted by the 5 most impactful miRNAs in MEITL (left) and EATL (right). Considering the 5 most impactful miRNAs, 73% and 36% of their mRNAs targets are shared by at least two of these miRNAs in MEITL and EATL respectively.

Supplemental Figure S12. Deconvolution analysis of RNA sequencing data. Each row represents a sample and shows the proportions of cell-type fractions as computed by the deconvolution algorithm Cibersort (colored by cell type).

Supplemental Figure S13. Methylation data. Unsupervised hierarchical clustering according to the 10'000 most variable CpGs (Euclidean distance). The presence of somatic mutations in genes associated to epigenetics (namely *SETD2, CREPBP, TET2, ARID1A, H3-3A, H3-3B, SMCHD1, KMT2D, EP300*) is annotated for each sample at the top of the heatmap (red = yes, grey = no, white = no information).

References

1. Roberti A, Dobay MP, Bisig B, Vallois D, Boechat C, Lanitis E, et al. Type II enteropathy-associated T-cell lymphoma features a unique genomic profile with highly recurrent SETD2 alterations. Nat Commun. 2016;7.
2. Veloza L, Cavalieri D, Missiaglia E, Ledoux-Pilon A, Bisig B, Pereira B, et al. Monomorphic epitheliotropic intestinal T-cell lymphoma comprises morphologic and genomic heterogeneity impacting outcome. Haematologica. 2023;108(1):181-95.
3. Swerdlow SH, Campo E, Pileri SA, Harris NL, Stein H, Siebert R, et al. The 2016 revision of the World Health Organization classification of lymphoid neoplasms. Blood. 2016;127(20):2375-90.
4. Bankhead P, Loughrey MB, Fernández JA, Dombrowski Y, McArt DG, Dunne PD, et al. QuPath: Open source software for digital pathology image analysis. Sci Rep. 2017;7(1):16878.
5. Cording S, Lhermitte L, Malamut G, Berrabah S, Trinquand A, Guegan N, et al. Oncogenetic landscape of lymphomagenesis in coeliac disease. Gut. 2022;71(3):497-508.
6. Koskela HL, Eldfors S, Ellonen P, van Adrichem AJ, Kuusanmäki H, Andersson EI, et al. Somatic STAT3 mutations in large granular lymphocytic leukemia. N Engl J Med. 2012;366(20):1905-13.
7. Milner JD, Vogel TP, Forbes L, Ma CA, Stray-Pedersen A, Niemela JE, et al. Early-onset lymphoproliferation and autoimmunity caused by germline STAT3 gain-of-function mutations. Blood. 2015;125(4):591-9.
8. Soderquist CR, Lewis SK, Gru AA, Vlad G, Williams ES, Hsiao S, et al. Immunophenotypic Spectrum and Genomic Landscape of Refractory Celiac Disease Type II. The American Journal of Surgical Pathology. 2021;45(7).

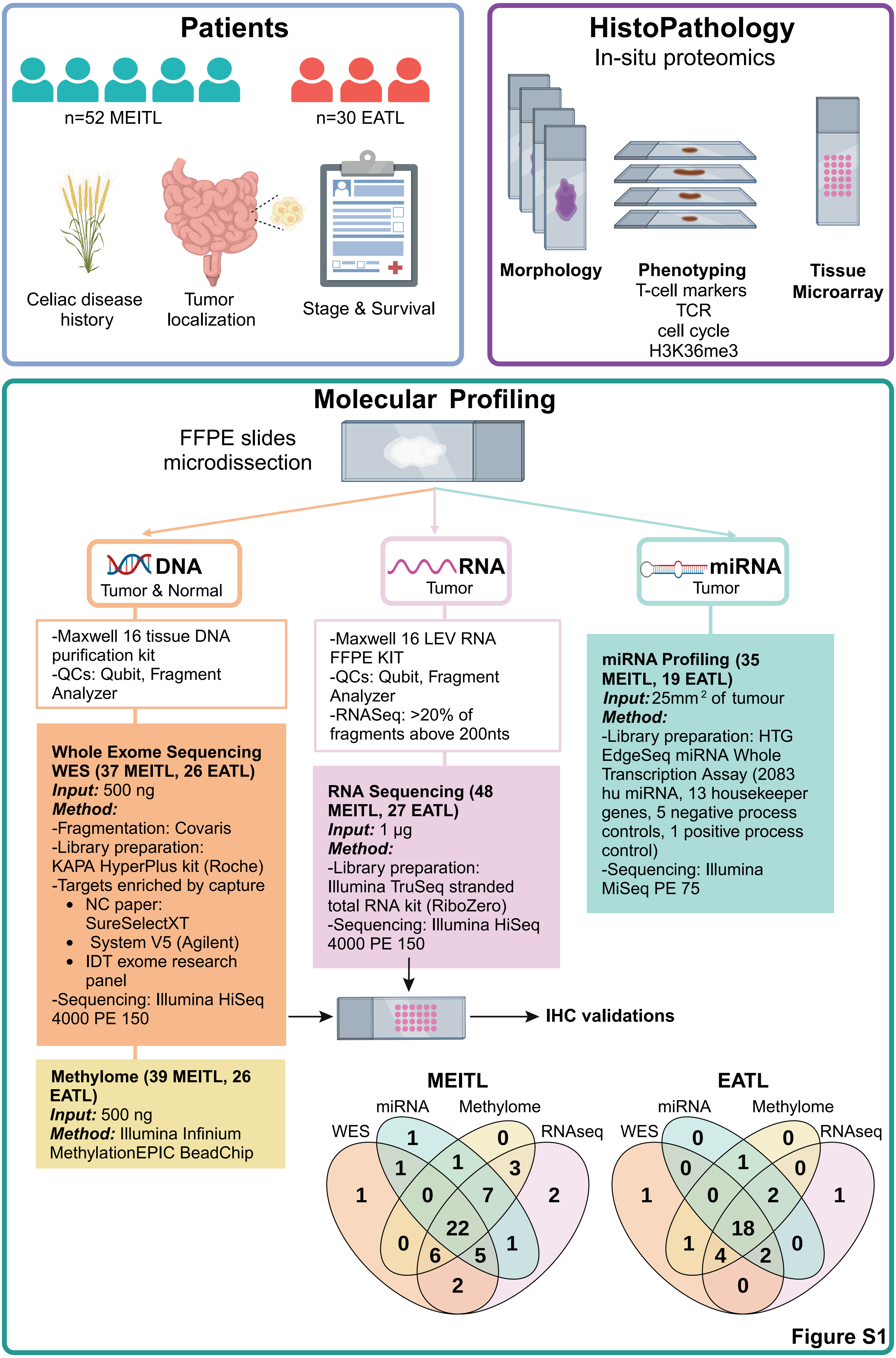
Patients
n=52 MEITL
n=30 EATL
Celiac disease history
Tumor localization
Stage & Survival
HistoPathology
In-situ proteomics
Morphology
Phenotyping
T-cell markers
TCR
cell cycle
H3K36me3
Tissue Microarray
Molecular Profiling
FFPE slides microdissection
DNA
Tumor & Normal
RNA
Tumor
miRNA
Tumor
-Maxwell 16 tissue DNA purification kit
-QCs: Qubit, Fragment Analyzer
Whole Exome Sequencing WES (37 MEITL, 26 EATL)
Input: 500 ng
Method:
-Fragmentation: Covaris
-Library preparation: KAPA HyperPlus kit (Roche)
-Targets enriched by capture
• NC paper: SureSelectXT
• System V5 (Agilent)
• IDT exome research panel
-Sequencing: Illumina HiSeq 4000 PE 150
Methylome (39 MEITL, 26 EATL)
Input: 500 ng
Method: Illumina Infinium MethylationEPIC BeadChip
-Maxwell 16 LEV RNA FFPE KIT
-QCs: Qubit, Fragment Analyzer
-RNASeq: >20% of fragments above 200nts
RNA Sequencing (48 MEITL, 27 EATL)
Input: 1 µg
Method:
-Library preparation: Illumina TruSeq stranded total RNA kit (RiboZero)
-Sequencing: Illumina HiSeq 4000 PE 150
miRNA Profiling (35 MEITL, 19 EATL)
Input: 25mm² of tumour
Method:
-Library preparation: HTG EdgeSeq miRNA Whole Transcription Assay (2083 hu miRNA, 13 housekeeper genes, 5 negative process controls, 1 positive process control)
-Sequencing: Illumina MiSeq PE 75
IHC validations
MEITL
miRNA
Methylome
WES
RNAseq
1 0 1 1 3 1 0 7 2 22 0 1 6 5 2
EATL
miRNA
Methylome
WES
RNAseq
0 0 0 1 0 1 0 2 1 18 1 0 4 2 0

Figure S1

A

Alleles associated with high risk of celiac disease	MEITL	EATL	p value
HLA-DQB1*02	14/48 (29%)	25/27 (93%)	****
HLA-DQA1*05	18/48 (37%)	24/27 (89%)	****
**Haplotypes associated with high risk of celiac disease**	**MEITL**	**EATL**	**p value**
HLA-DQ2.5 (HLA-DQA1*05/HLA-DQB1*02)	10/48 (20%)	23/27 (85%)	****
HLA-DQB1*02 homozygozity	3/48 (6%)	8/27 (30%)	*
HLA-DQ8 (HLA-DQA1*03/HLA-DQB1*0302)	2/48 (4%)	0	ns
Any of the celiac disease HLA high risk haplotypes	12/48 (25%)	23/27 (85%)	****
**Haplotypes associated with low risk of celiac disease**	**MEITL**	**EATL**	**p value**
HLA-DQ2.x (HLA-DQB1*02/any DQA1 different from DQA1*05)	4/48 (8%)	2/27 (7%)	ns
HLA-DQX.5 (HLA-DQA1*05/any DQB1 different from *02 and *0302)	5/48 (10%)	1/27 (4%)	ns

B

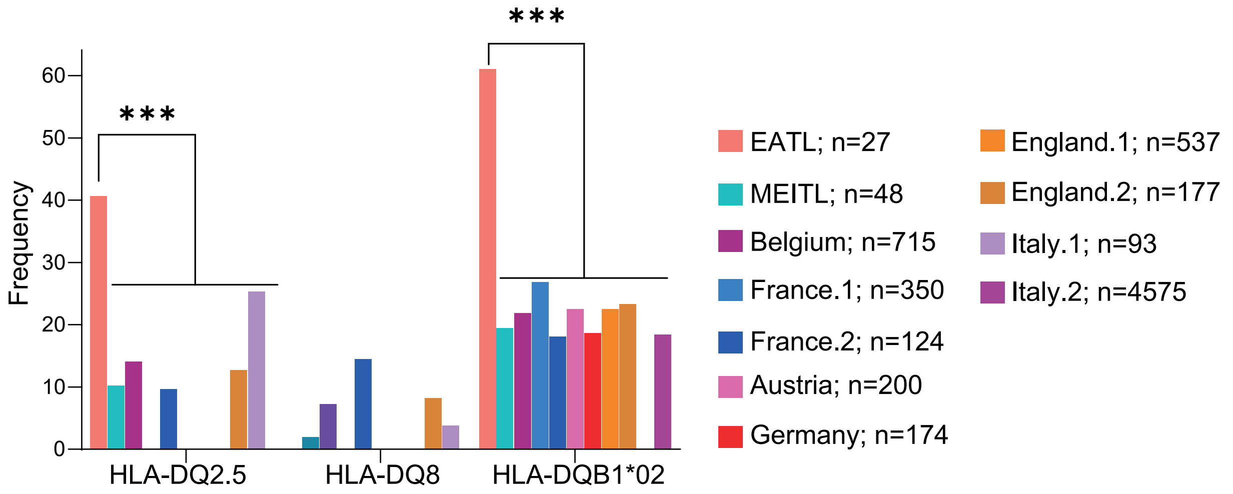

Figure S2

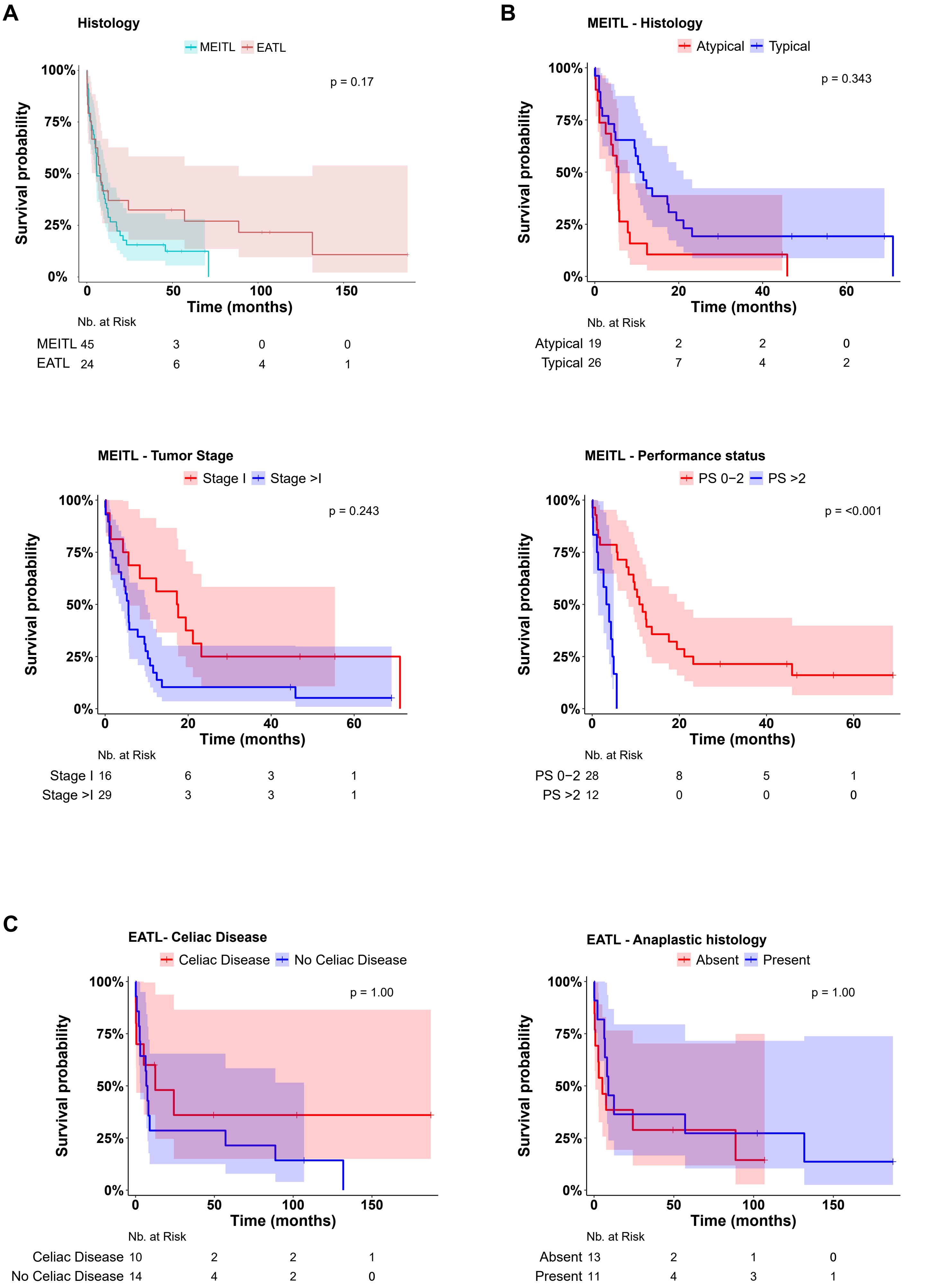
A
Histology
MEITL
EATL
p = 0.17
Survival probability
100%
75%
50%
25%
0%
0
50
100
150
Time (months)
Nb. at Risk
MEITL 45 3 0 0
EATL 24 6 4 1
B
MEITL - Histology
Atypical
Typical
p = 0.343
Survival probability
100%
75%
50%
25%
0%
0
20
40
60
Time (months)
Nb. at Risk
Atypical 19 2 2 0
Typical 26 7 4 2
MEITL - Tumor Stage
Stage I
Stage >I
p = 0.243
Survival probability
100%
75%
50%
25%
0%
0
20
40
60
Time (months)
Nb. at Risk
Stage I 16 6 3 1
Stage >I 29 3 3 1
MEITL - Performance status
PS 0−2
PS >2
p = <0.001
Survival probability
100%
75%
50%
25%
0%
0
20
40
60
Time (months)
Nb. at Risk
PS 0−2 28 8 5 1
PS >2 12 0 0 0
C
EATL- Celiac Disease
Celiac Disease
No Celiac Disease
p = 1.00
Survival probability
100%
75%
50%
25%
0%
0
50
100
150
Time (months)
Nb. at Risk
Celiac Disease 10 2 2 1
No Celiac Disease 14 4 2 0
EATL - Anaplastic histology
Absent
Present
p = 1.00
Survival probability
100%
75%
50%
25%
0%
0
50
100
150
Time (months)
Nb. at Risk
Absent 13 2 1 0
Present 11 4 3 1

Figure S3

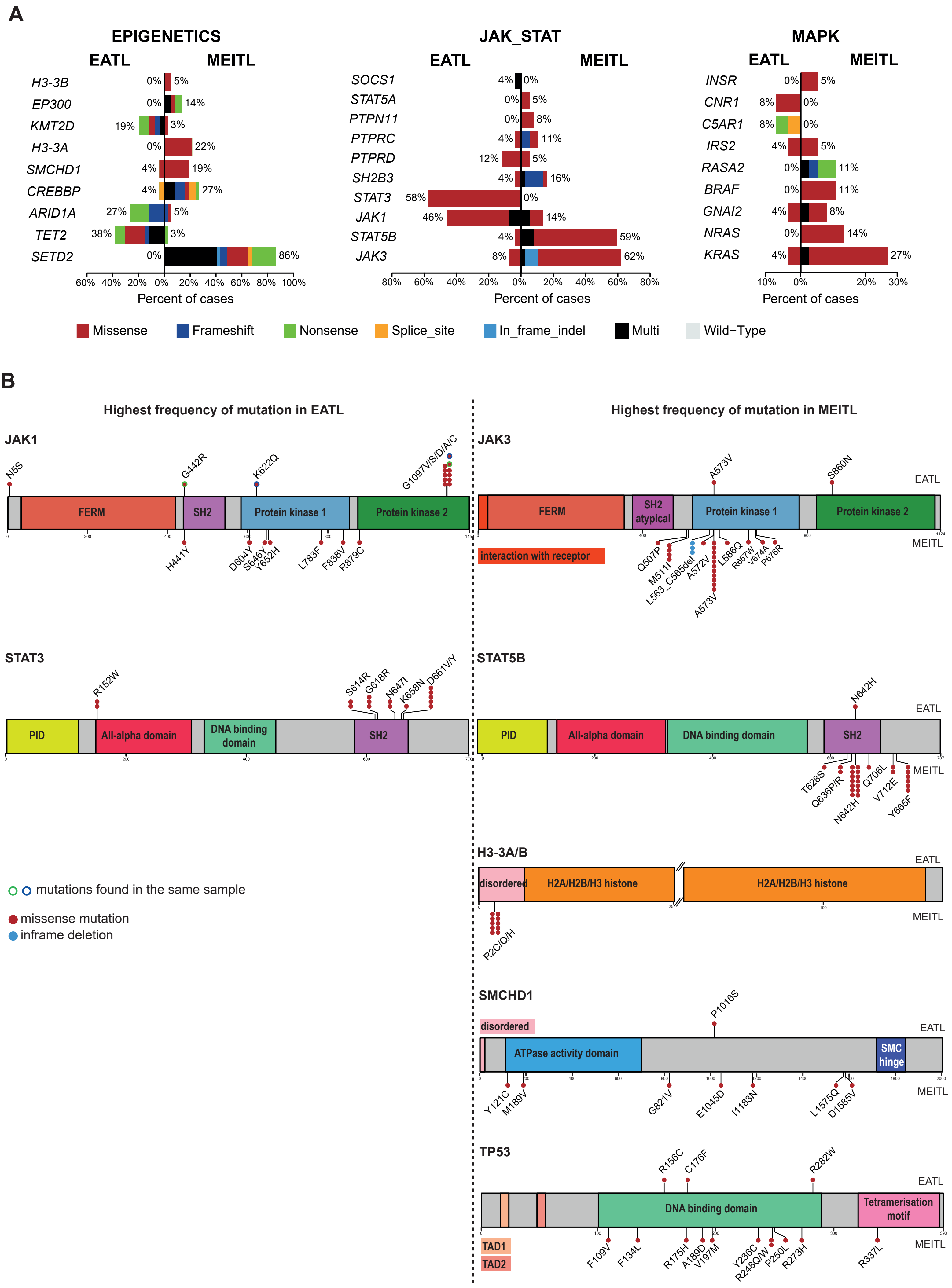
A
EPIGENETICS
EATL
MEITL
H3-3B
EP300
KMT2D
H3-3A
SMCHD1
CREBBP
ARID1A
TET2
SETD2
Percent of cases
JAK_STAT
SOCS1
STAT5A
PTPN11
PTPRC
PTPRD
SH2B3
STAT3
JAK1
STAT5B
JAK3
MAPK
INSR
CNR1
C5AR1
IRS2
RASA2
BRAF
GNAI2
NRAS
KRAS
Missense
Frameshift
Nonsense
Splice_site
In_frame_indel
Multi
Wild−Type
B
Highest frequency of mutation in EATL
Highest frequency of mutation in MEITL
JAK1
FERM
SH2
Protein kinase 1
Protein kinase 2
JAK3
SH2 atypical
interaction with receptor
STAT3
PID
All-alpha domain
DNA binding domain
STAT5B
H3-3A/B
disordered
H2A/H2B/H3 histone
SMCHD1
ATPase activity domain
SMC hinge
TP53
Tetramerisation motif
TAD1
TAD2
mutations found in the same sample
missense mutation
inframe deletion

Figure S4

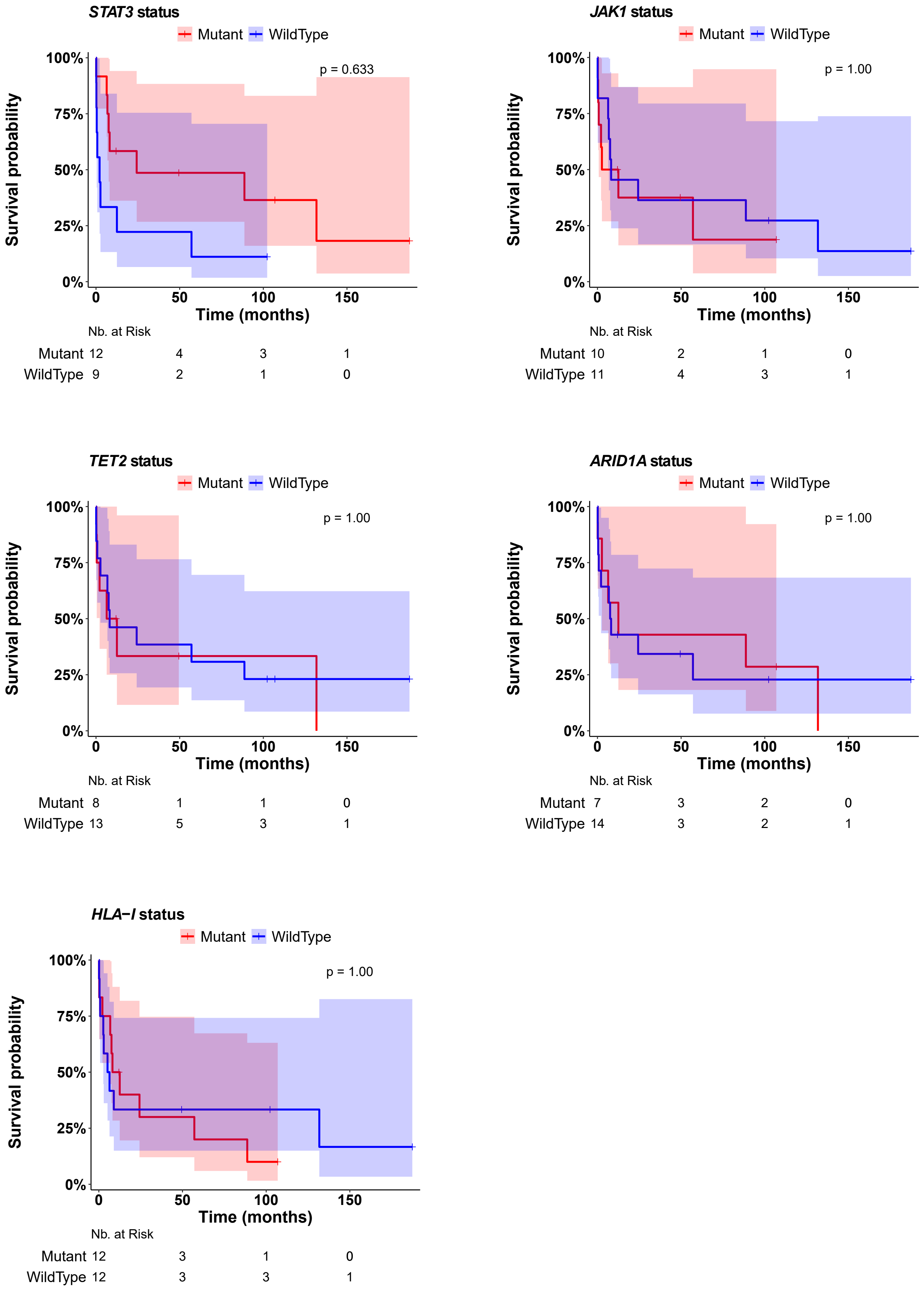
STAT3 status
Mutant
WildType
p = 0.633
Survival probability
100%
75%
50%
25%
0%
0
50
100
150
Time (months)
Nb. at Risk
Mutant 12 4 3 1
WildType 9 2 1 0
JAK1 status
Mutant
WildType
p = 1.00
Survival probability
Time (months)
Nb. at Risk
Mutant 10 2 1 0
WildType 11 4 3 1
TET2 status
Mutant
WildType
p = 1.00
Survival probability
Time (months)
Nb. at Risk
Mutant 8 1 1 0
WildType 13 5 3 1
ARID1A status
Mutant
WildType
p = 1.00
Survival probability
Time (months)
Nb. at Risk
Mutant 7 3 2 0
WildType 14 3 2 1
HLA−I status
Mutant
WildType
p = 1.00
Survival probability
Time (months)
Nb. at Risk
Mutant 12 3 1 0
WildType 12 3 3 1

Figure S5

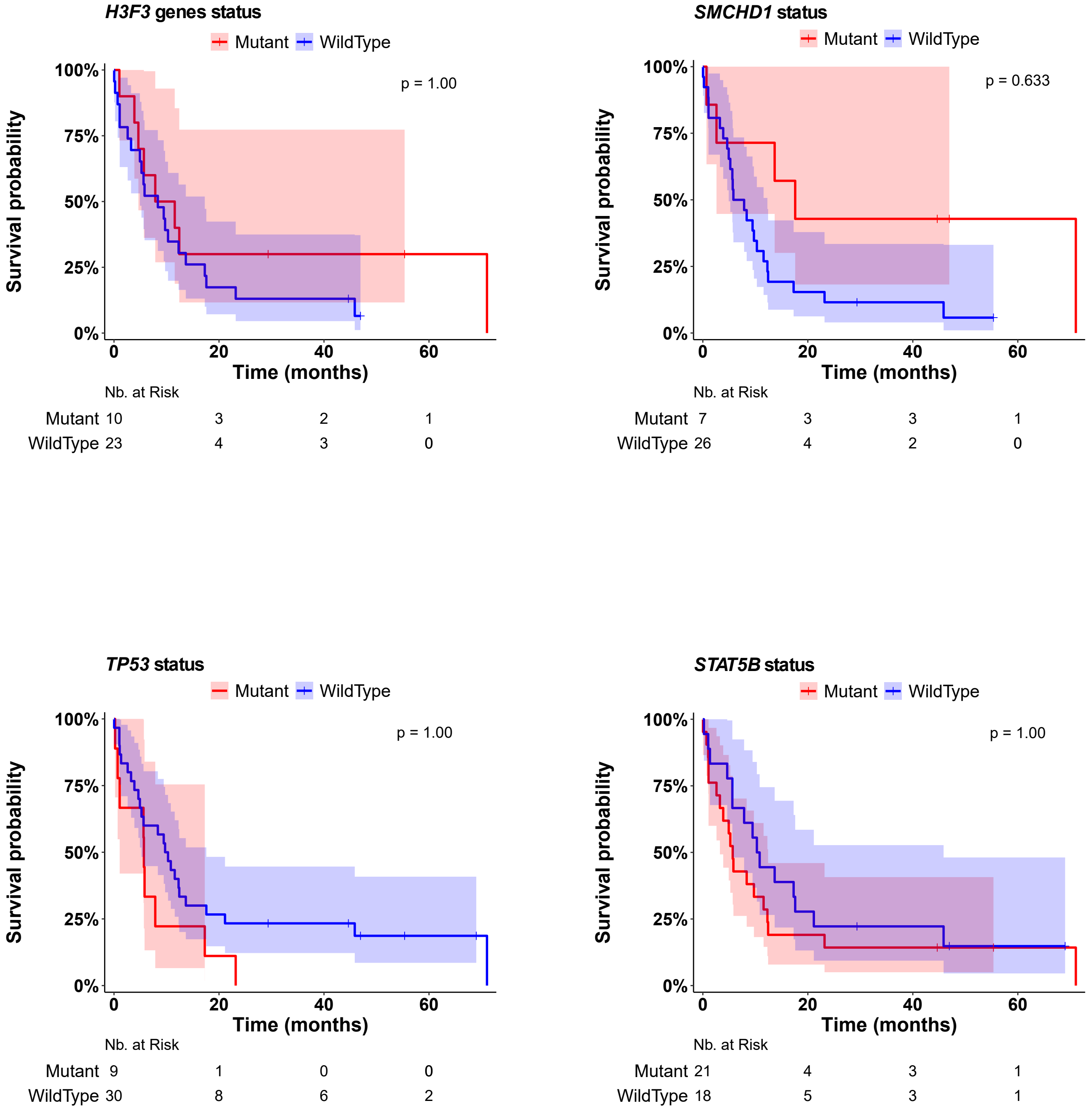
H3F3 genes status
Mutant
WildType
p = 1.00
Survival probability
100%
75%
50%
25%
0%
0
20
40
60
Time (months)
Nb. at Risk
Mutant 10 3 2 1
WildType 23 4 3 0
SMCHD1 status
Mutant
WildType
p = 0.633
Survival probability
Time (months)
Nb. at Risk
Mutant 7 3 3 1
WildType 26 4 2 0
TP53 status
Mutant
WildType
p = 1.00
Survival probability
Time (months)
Nb. at Risk
Mutant 9 1 0 0
WildType 30 8 6 2
STAT5B status
Mutant
WildType
p = 1.00
Survival probability
Time (months)
Nb. at Risk
Mutant 21 4 3 1
WildType 18 5 3 1

Figure S6

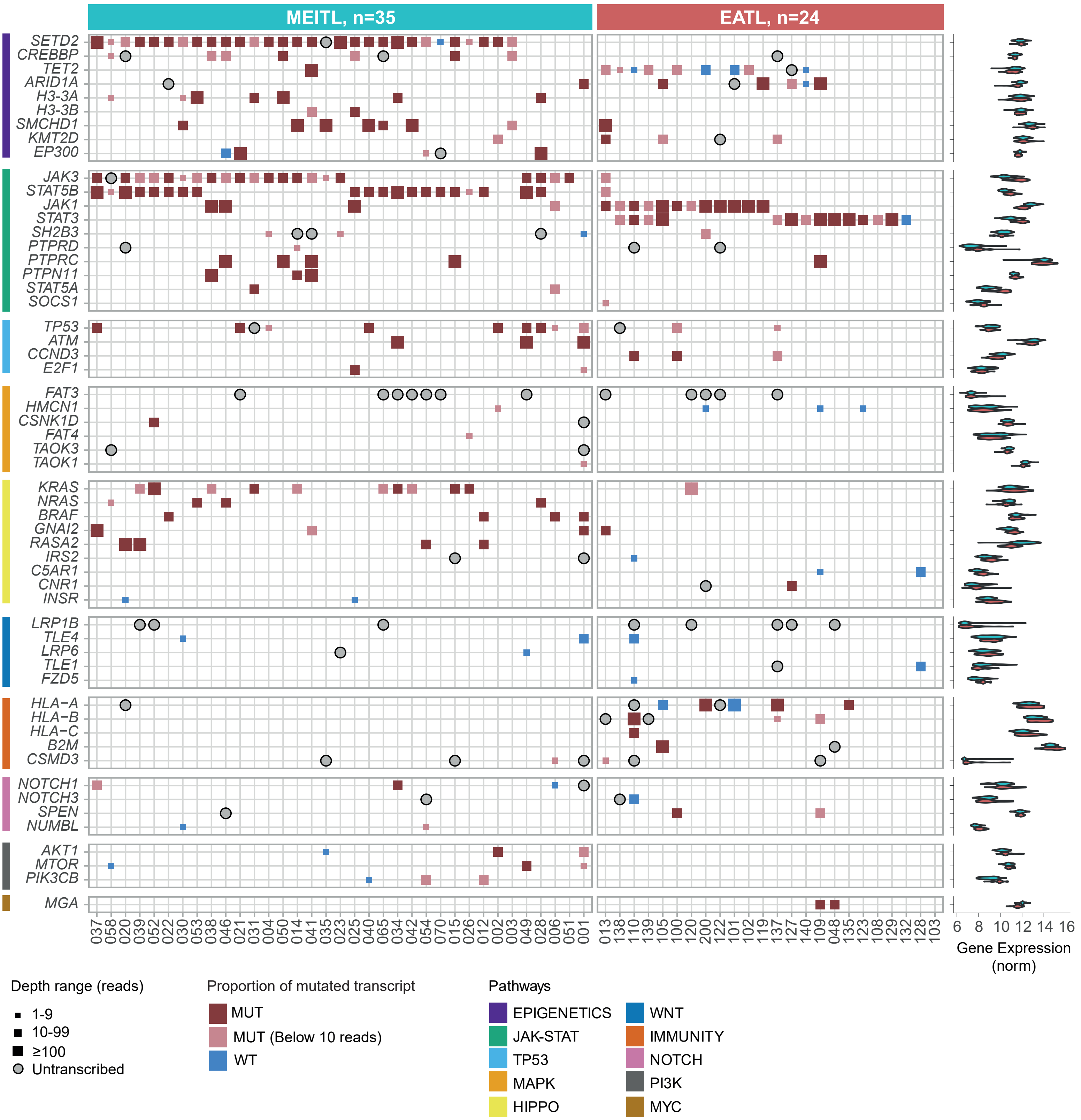

MEITL, n=35
EATL, n=24
SETD2
CREBBP
TET2
ARID1A
H3-3A
H3-3B
SMCHD1
KMT2D
EP300
JAK3
STAT5B
JAK1
STAT3
SH2B3
PTPRD
PTPRC
PTPN11
STAT5A
SOCS1
TP53
ATM
CCND3
E2F1
FAT3
HMCN1
CSNK1D
FAT4
TAOK3
TAOK1
KRAS
NRAS
BRAF
GNAI2
RASA2
IRS2
C5AR1
CNR1
INSR
LRP1B
TLE4
LRP6
TLE1
FZD5
HLA−A
HLA−B
HLA−C
B2M
CSMD3
NOTCH1
NOTCH3
SPEN
NUMBL
AKT1
MTOR
PIK3CB
MGA
037 058 020 039 052 022 030 053 038 046 021 031 004 050 014 041 035 023 025 040 065 034 042 054 070 015 026 012 002 003 049 028 006 051 001
013 138 110 139 105 100 120 200 122 101 102 119 137 127 140 109 048 135 123 108 129 132 128 103
6 8 10 12 14 16
Gene Expression (norm)
Depth range (reads)
1-9
10-99
≥100
Untranscribed
Proportion of mutated transcript
MUT
MUT (Below 10 reads)
WT
Pathways
EPIGENETICS
JAK-STAT
TP53
MAPK
HIPPO
WNT
IMMUNITY
NOTCH
PI3K
MYC

Figure S7

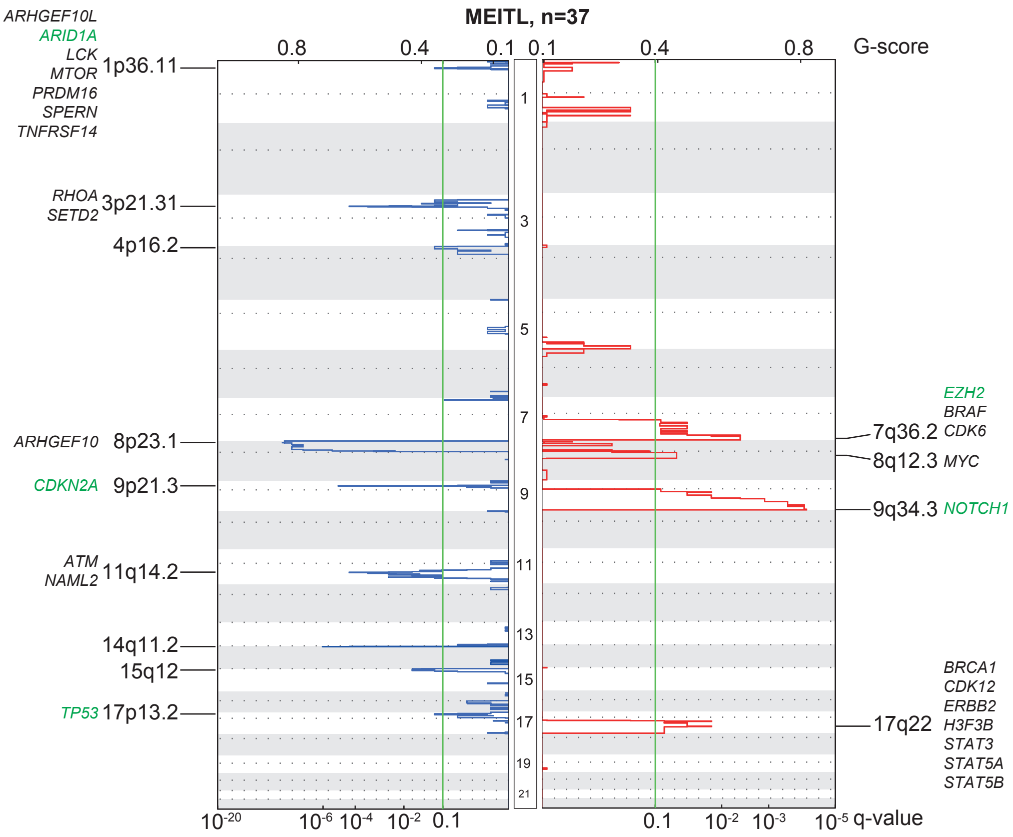

MEITL, n=37
0.8
0.4
0.1
0.1
0.4
0.8
G-score
ARHGEF10L
ARID1A
LCK
MTOR
PRDM16
SPERN
TNFRSF14
1p36.11
RHOA
SETD2
3p21.31
4p16.2
ARHGEF10
8p23.1
CDKN2A
9p21.3
ATM
NAML2
11q14.2
14q11.2
15q12
TP53
17p13.2
EZH2
BRAF
7q36.2
CDK6
8q12.3
MYC
9q34.3
NOTCH1
BRCA1
CDK12
ERBB2
17q22
H3F3B
STAT3
STAT5A
STAT5B
1
3
5
7
9
11
13
15
17
19
21
10-20
10-6
10-4
10-2
0.1
0.1
10-2
10-3
10-5
q-value

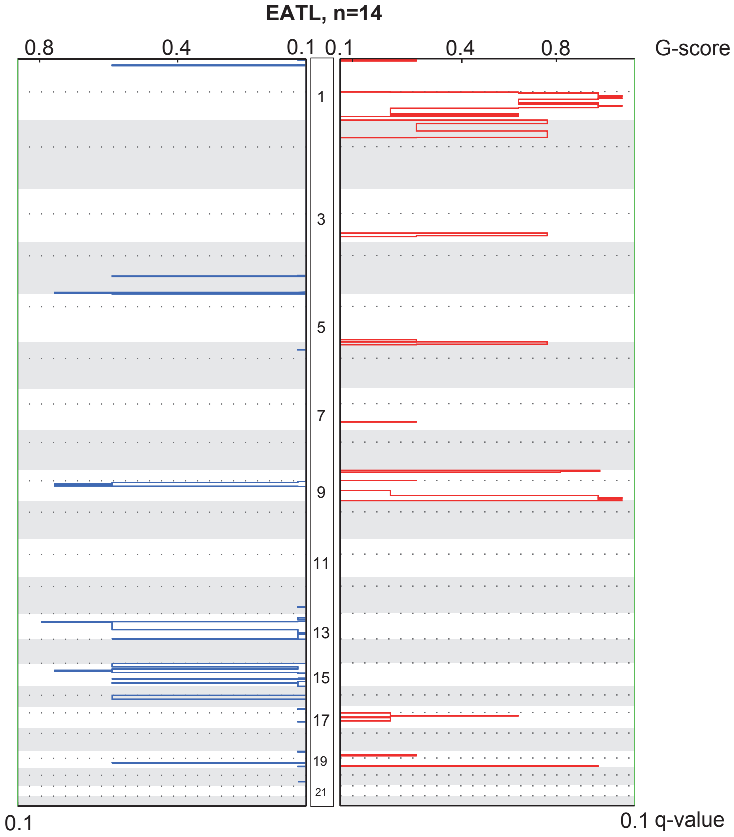

EATL, n=14
0.8
0.4
0.1
0.1
0.4
0.8
G-score
1
3
5
7
9
11
13
15
17
19
21
0.1
0.1
q-value

Figure S8

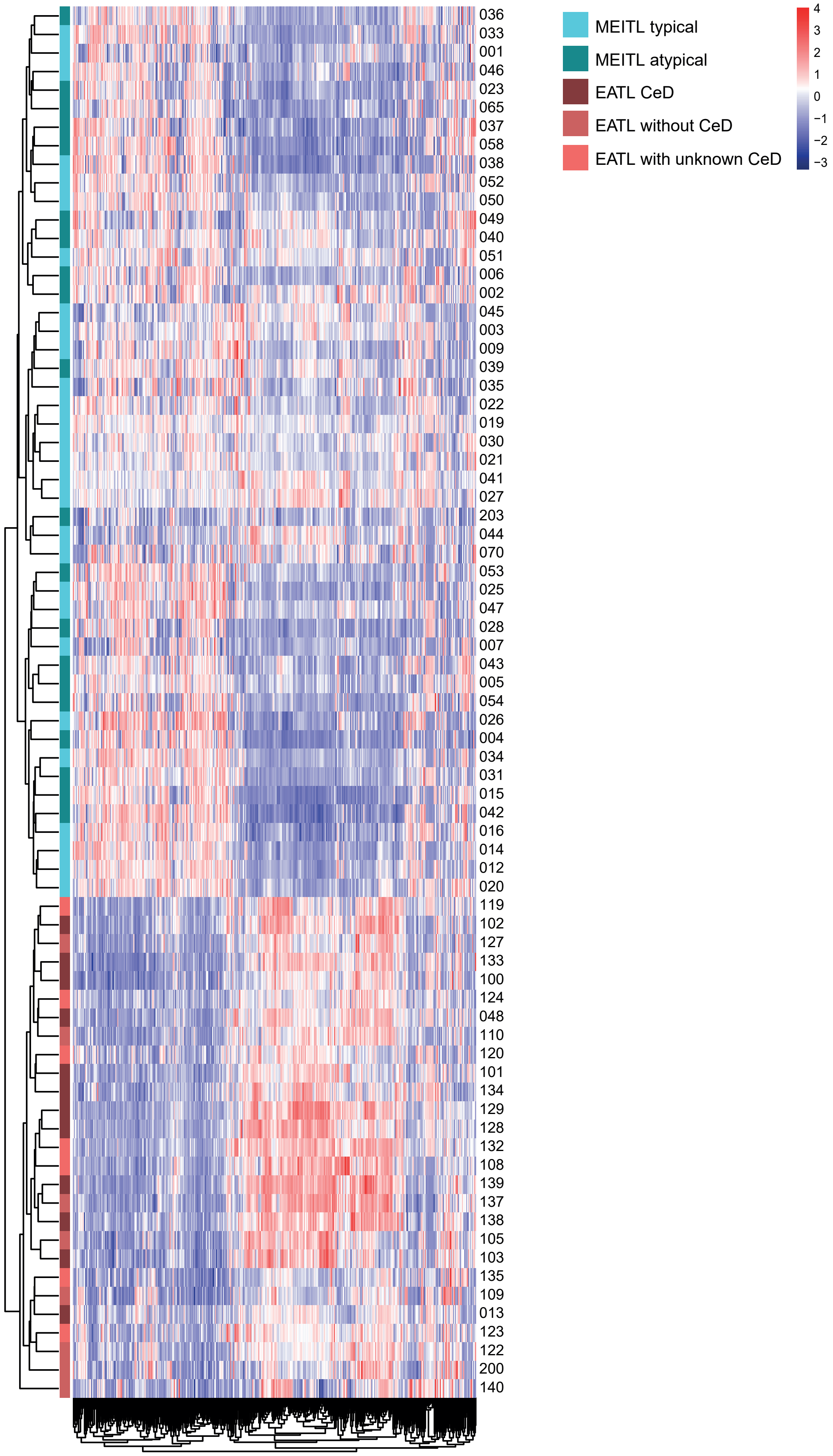
036
033
001
046
023
065
037
058
038
052
050
049
040
051
006
002
045
003
009
039
035
022
019
030
021
041
027
203
044
070
053
025
047
028
007
043
005
054
026
004
034
031
015
042
016
014
012
020
119
102
127
133
100
124
048
110
120
101
134
129
128
132
108
139
137
138
105
103
135
109
013
123
122
200
140
MEITL typical
MEITL atypical
EATL CeD
EATL without CeD
EATL with unknown CeD
4
3
2
1
0
−1
−2
−3

Figure S9

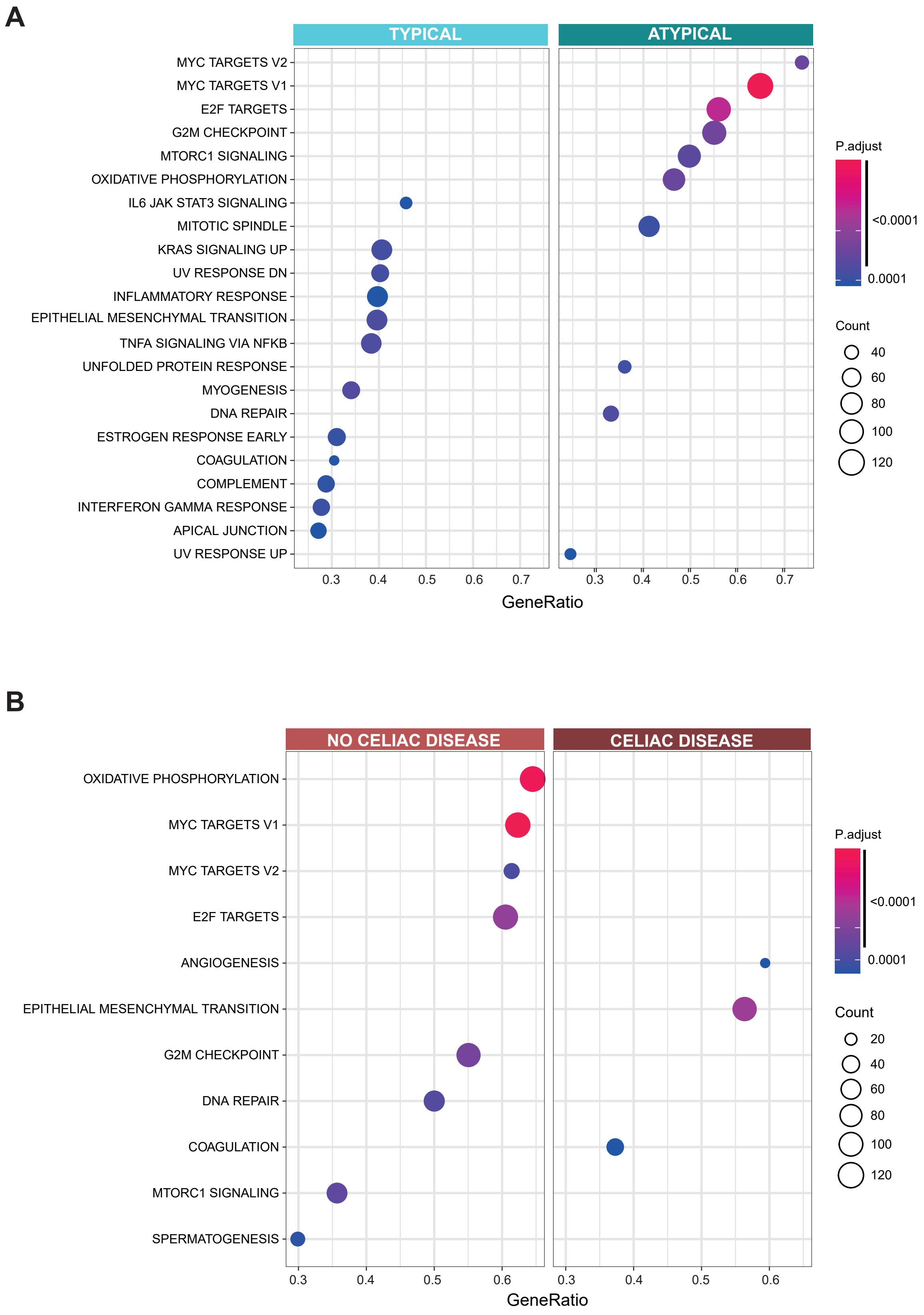
A
TYPICAL
ATYPICAL
MYC TARGETS V2
MYC TARGETS V1
E2F TARGETS
G2M CHECKPOINT
MTORC1 SIGNALING
OXIDATIVE PHOSPHORYLATION
IL6 JAK STAT3 SIGNALING
MITOTIC SPINDLE
KRAS SIGNALING UP
UV RESPONSE DN
INFLAMMATORY RESPONSE
EPITHELIAL MESENCHYMAL TRANSITION
TNFA SIGNALING VIA NFKB
UNFOLDED PROTEIN RESPONSE
MYOGENESIS
DNA REPAIR
ESTROGEN RESPONSE EARLY
COAGULATION
COMPLEMENT
INTERFERON GAMMA RESPONSE
APICAL JUNCTION
UV RESPONSE UP
0.3 0.4 0.5 0.6 0.7
GeneRatio
P.adjust
<0.0001
0.0001
Count
40
60
80
100
120
B
NO CELIAC DISEASE
CELIAC DISEASE
OXIDATIVE PHOSPHORYLATION
MYC TARGETS V1
MYC TARGETS V2
E2F TARGETS
ANGIOGENESIS
EPITHELIAL MESENCHYMAL TRANSITION
G2M CHECKPOINT
DNA REPAIR
COAGULATION
MTORC1 SIGNALING
SPERMATOGENESIS
0.3 0.4 0.5 0.6
GeneRatio
P.adjust
<0.0001
0.0001
Count
20
40
60
80
100
120

Figure S10

A

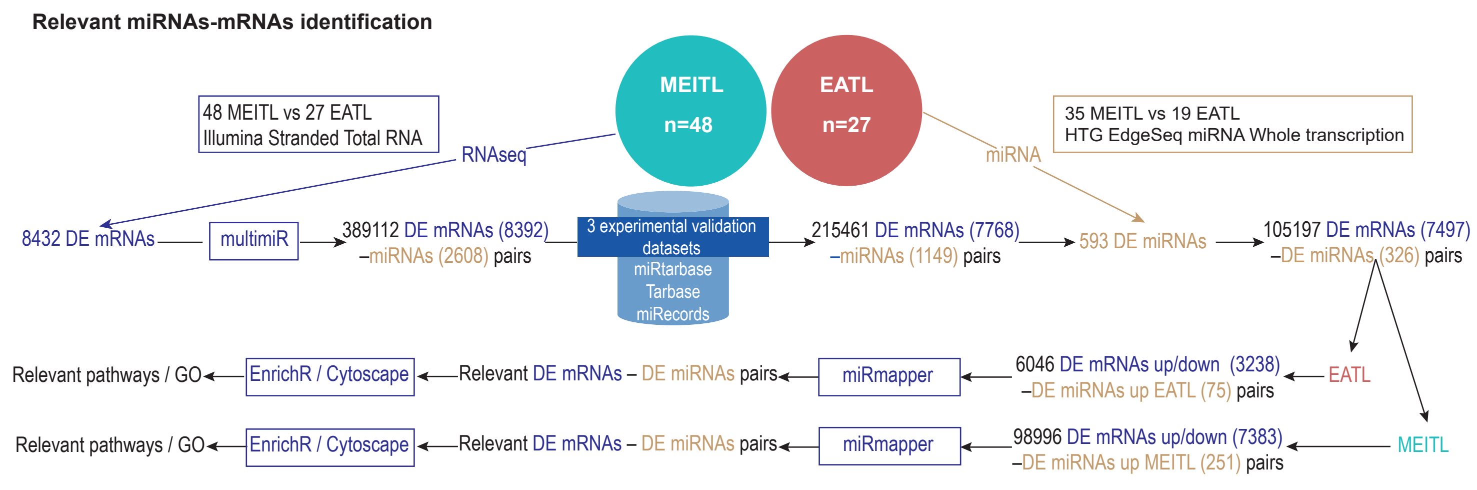

B

Top 5 most impactful miRNAs on GEP in MEITL and EATL

MEITL (# of mRNAs targets)	EATL (# of mRNAs targets)
miR-27a-3p (3095)	miR-21-3p (1051)
miR-15-5p (1559)	miR-503-5p (485)
miR-107 (1907)	miR-671-5p (511)
miR-103a-2p (1780)	miR-939-5p (291)
miR-146a-5p (2112)	miR-485-3p (252)

EnrichR → Cytoscape

Pathways impacted by the most impactful miRNAs

Pathways	MEITL	EATL
**PI3K-AKT-mTOR**	Down	Up
**Cytokines signaling**	Down	Up
**TLR/NF-kB**	Down	Up
**EMT**	Up	Down
**Cell Cycle**	Up	Down

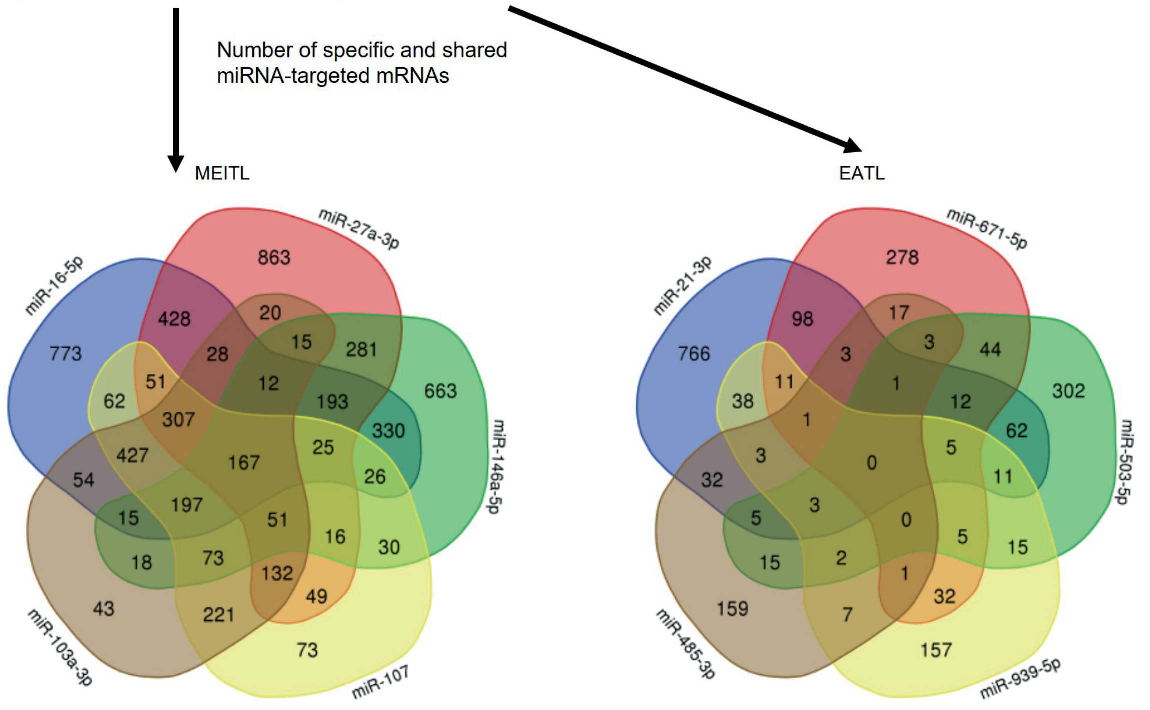

Figure S11

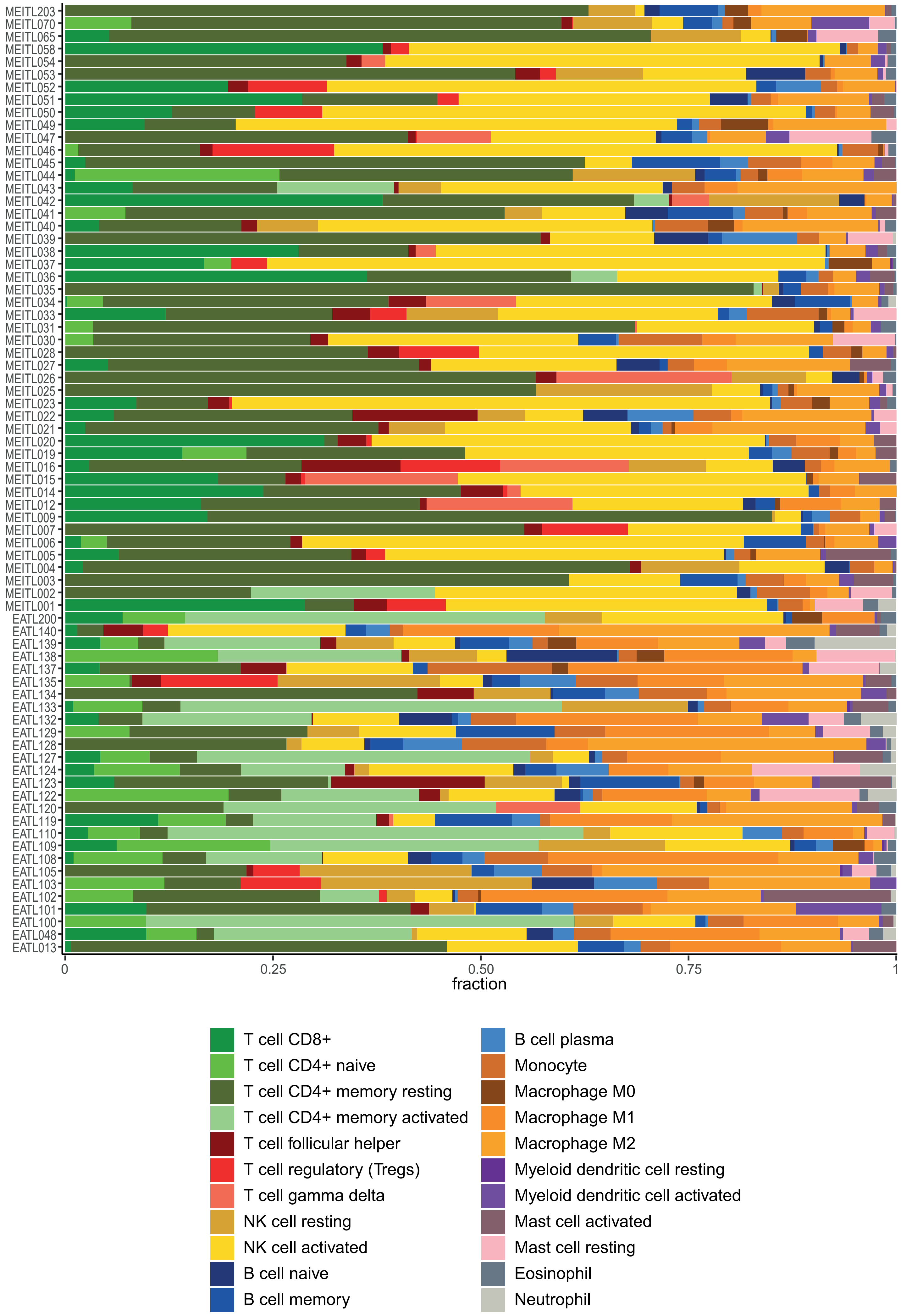
MEITL203
MEITL070
MEITL065
MEITL058
MEITL054
MEITL053
MEITL052
MEITL051
MEITL050
MEITL049
MEITL047
MEITL046
MEITL045
MEITL044
MEITL043
MEITL042
MEITL041
MEITL040
MEITL039
MEITL038
MEITL037
MEITL036
MEITL035
MEITL034
MEITL033
MEITL031
MEITL030
MEITL028
MEITL027
MEITL026
MEITL025
MEITL023
MEITL022
MEITL021
MEITL020
MEITL019
MEITL016
MEITL015
MEITL014
MEITL012
MEITL009
MEITL007
MEITL006
MEITL005
MEITL004
MEITL003
MEITL002
MEITL001
EATL200
EATL140
EATL139
EATL138
EATL137
EATL135
EATL134
EATL133
EATL132
EATL129
EATL128
EATL127
EATL124
EATL123
EATL122
EATL120
EATL119
EATL110
EATL109
EATL108
EATL105
EATL103
EATL102
EATL101
EATL100
EATL048
EATL013
0
0.25
0.50
0.75
1
fraction
T cell CD8+
T cell CD4+ naive
T cell CD4+ memory resting
T cell CD4+ memory activated
T cell follicular helper
T cell regulatory (Tregs)
T cell gamma delta
NK cell resting
NK cell activated
B cell naive
B cell memory
B cell plasma
Monocyte
Macrophage M0
Macrophage M1
Macrophage M2
Myeloid dendritic cell resting
Myeloid dendritic cell activated
Mast cell activated
Mast cell resting
Eosinophil
Neutrophil

Figure S12

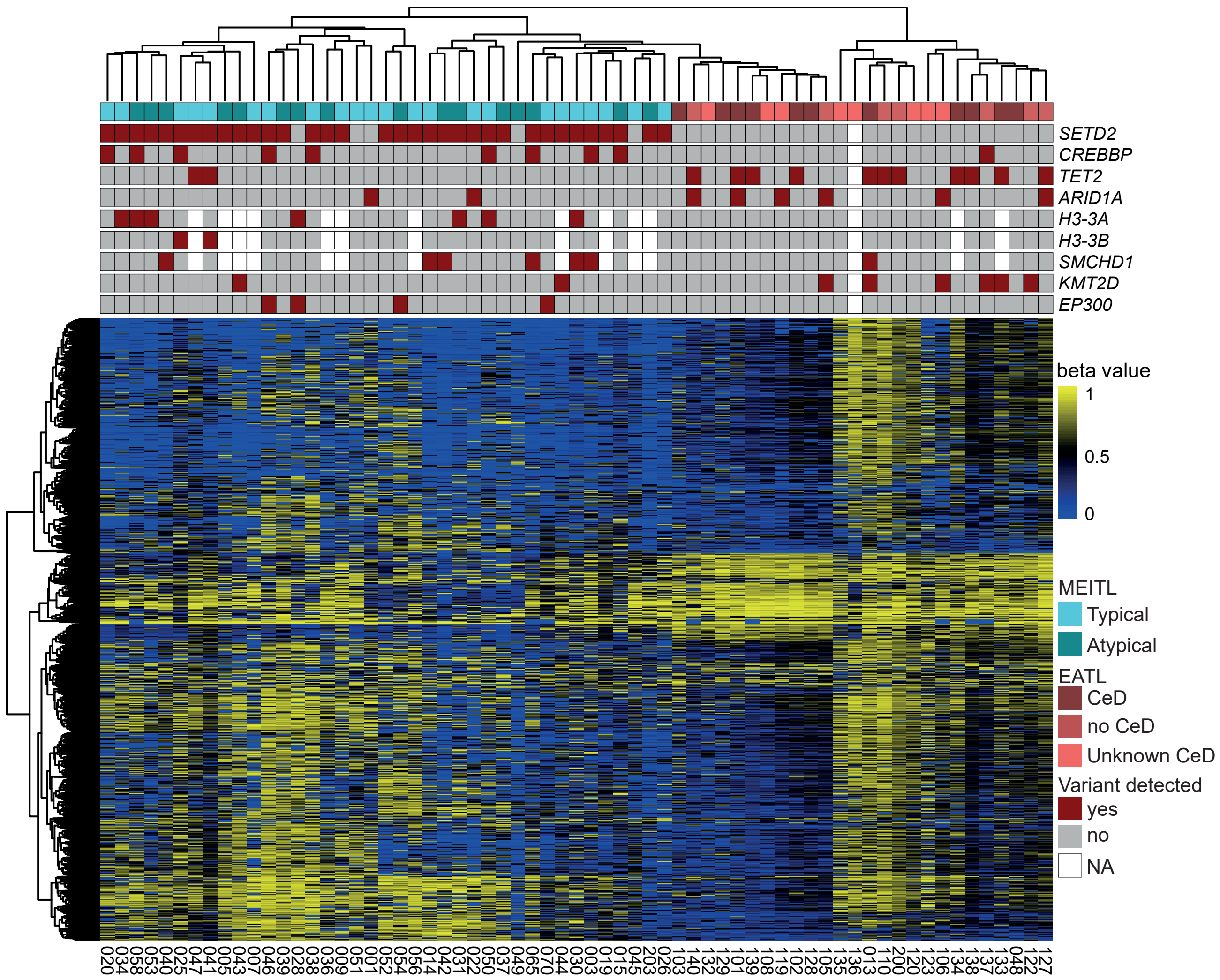
SETD2
CREBBP
TET2
ARID1A
H3-3A
H3-3B
SMCHD1
KMT2D
EP300
beta value
1
0.5
0
MEITL
Typical
Atypical
EATL
CeD
no CeD
Unknown CeD
Variant detected
yes
no
NA

Figure S13
